# Factors affecting the use of antibiotics and antiseptics to prevent maternal infection at birth: A global mixed-methods systematic review

**DOI:** 10.1371/journal.pone.0272982

**Published:** 2022-09-01

**Authors:** Katherine E. Eddy, Rana Islamiah Zahroh, Meghan A. Bohren, Mercedes Bonet, Caroline S. E. Homer, Joshua P. Vogel

**Affiliations:** 1 Maternal, Child and Adolescent Health Program, Burnet Institute, Melbourne, Australia; 2 Gender and Women’s Health Unit, Centre for Health Equity, School of Population and Global Health, University of Melbourne, Melbourne, Australia; 3 UNDP/UNFPA/UNICEF/WHO/World Bank Special Programme of Research, Development and Research Training in Human Reproduction (HRP), Department of Sexual and Reproductive Health and Research, World Health Organization, Geneva, Switzerland; Johns Hopkins University Bloomberg School of Public Health, UNITED STATES

## Abstract

**Background:**

Over 10% of maternal deaths annually are due to sepsis. Prophylactic antibiotics and antiseptic agents are critical interventions to prevent maternal peripartum infections. We conducted a mixed-method systematic review to better understand factors affecting the use of prophylactic antibiotics and antiseptic agents to prevent peripartum infections.

**Methods:**

We searched MEDLINE, EMBASE, Emcare, CINAHL, Global Health, Global Index Medicus, and Maternity and Infant Care for studies published between 1 January 1990 and 27 May 2022. We included primary qualitative, quantitative, and mixed-methods studies that focused on women, families, and healthcare providers’ perceptions and experiences of prophylactic antibiotic and antiseptics during labour and birth in health facilities. There were no language restrictions. We used a thematic synthesis approach for qualitative evidence and GRADE-CERQual approach for assessing confidence in these review findings. Quantitative study results were mapped to the qualitative findings and reported narratively.

**Results:**

We included 19 studies (5 qualitative, 12 quantitative and 2 mixed-methods studies), 16 relating to antibiotics, 2 to antiseptic use, and 1 study to both antibiotic and antiseptic use. Most related to providers’ perspectives and were conducted in high-income countries. Key themes on factors affecting antibiotic use were providers’ beliefs about benefits and harms, perceptions of women’s risk of infection, regimen preferences and clinical decision-making processes. Studies on antiseptic use explored women’s perceptions of vaginal cleansing, and provider’s beliefs about benefits and the usefulness of guidelines.

**Conclusion:**

We identified a range of factors affecting how providers use prophylactic antibiotics at birth, which can undermine implementation of clinical guidelines. There were insufficient data for low-resource settings, women’s perspectives, and regarding use of antiseptics, highlighting the need for further research in these areas. Implications for practice include that interventions to improve prophylactic antibiotic use should take account of local environments and perceived infection risk and ensure contextually relevant guidance.

## Introduction

All women who give birth are at risk of developing peripartum infection, which can lead to sepsis, septic shock and death. Sepsis accounts for over 10% of the 295,000 maternal deaths that occur globally each year [1, 2]. Women who survive peripartum infections are prone to longer-term complications, including chronic pain and secondary infertility [3]. Babies born to women experiencing infection are at risk of intrapartum asphyxia or neonatal infection, increasing the likelihood of preterm birth and neonatal death [4, 5]. The risk of infection is higher for women undergoing caesarean section, increased at least five-fold compared to women who have a vaginal birth [6, 7], and is also higher for women who experience more than five vaginal examinations, manual removal of the placenta, instrumental vaginal birth (vacuum or forceps) or obstetric complications [6, 8]. Women who have pre-existing anaemia, obesity or diabetes are also at increased risk of infection [1, 9].

Appropriate use of topical antiseptic agents and prophylactic antibiotics are critical elements of good-quality maternity care and can prevent peripartum infections from occurring [8]. In some subgroups of women who are at higher risk of infection or undergoing a procedure, prophylactic antibiotic administration ensures a sufficient concentration of antimicrobial agents in serum and tissue is present in order to prevent an infection from establishing itself. Similarly, topical application of antiseptic agents (such as chlorhexidine or iodine prior to Caesarean section) reduces the number of microbes present on the skin, thus reducing the likelihood of post-procedural infection. In 2015, the World Health Organization (WHO) recommended that prophylactic antibiotics should be used for women experiencing caesarean section, preterm prelabour rupture of membranes, or manual removal of placenta [3]. WHO also recommends antiseptics for perioperative skin preparation and vaginal cleansing for women undergoing caesarean section to prevent post-operative maternal infectious morbidities [3]. In 2021, WHO revalidated its prior recommendations on prophylactic antibiotics, skin preparation and vaginal cleansing for caesarean section, indicating that the evidence base on benefits and harms of interventions has not changed substantively in the past several years [10–12].

Despite clear evidence of benefit, antibiotic and antiseptic prophylaxis are often misused in maternity care settings [3, 13]. Unnecessary overuse of antibiotics can cause avoidable harm to women and babies through side effects, and more broadly by increasing antimicrobial resistance [14–17]. Conversely, in some settings appropriate antimicrobial prevention interventions are underutilised; one study on peripartum antibiotic use across 29 countries found a third of maternity care facilities had poor coverage of antibiotic prophylaxis for women undergoing caesarean section [18]. Understanding the factors affecting peripartum antibiotic and antiseptic use from the perspectives of women and healthcare providers is essential to encourage their safe and appropriate use, and understand potential explanations for underuse or misuse that can be addressed through behaviour change [1].

While previous individual studies have explored providers’ and women’s perspectives on antibiotic prophylaxis and antiseptic agents for preventing infection at birth, to date no systematic reviews have synthesised this evidence across multiple contexts [19, 20]. We therefore aimed to synthesise evidence on factors affecting the use of prophylactic antibiotics and antiseptic agents for the prevention of peripartum infection during labour and birth, from the perspectives and experiences of women, partners, families, and healthcare providers.

## Methods

This mixed-methods systematic review was registered with the International Prospective Register of Systematic Reviews (PROSPERO, CRD42020191746), reported according to the Preferred Reporting Items for Systematic Reviews and Meta-Analyses (PRISMA) checklist (S1 Appendix), and guided by the Cochrane Effective Practice and Organisation of Care template for conducting qualitative evidence synthesis [21]. There was no patient or public involvement.

### Eligibility criteria

The review scope was defined using an adapted setting, perspective, intervention, comparison, and evaluation (SPICE) framework [22]. We sought the perspective (P) of women giving birth, their partners and families, and healthcare providers in healthcare facilities globally (S). The interventions (I) were the use of antibiotics and antiseptics for prevention of infection during labour and birth, there was no comparison (C), and we were specifically interested in their perspectives and experiences on factors affecting use of the interventions (E) [23].

Primary qualitative, quantitative, and mixed-methods studies were eligible. For the qualitative component, studies that used both a qualitative data collection method (e.g. focus group discussions, individual interviews, observation, diaries, document analysis, open-ended survey questions) and qualitative data analysis (e.g. thematic analysis, framework analysis, grounded theory) were eligible. For the quantitative component, primary studies using an observational or interventional design (including randomised controlled trials, cohort studies, cross-sectional studies) were eligible. Mixed-methods studies were also eligible. We excluded other article types such as case reports, case series, letters, editorials, commentaries, reviews, study protocols, and conference abstracts. One study has been classified as “awaiting classification”, as no full text could be retrieved.

There was no restriction on language or country of publication. Full texts of studies published in languages other than English were translated using freely available online software (Google Translate). We included studies pertaining to any level of healthcare facility (e.g. hospitals, clinics, and primary healthcare settings). The timeframe of interest was the time from admission for childbirth until the woman’s discharge from the facility, i.e. the peripartum period during which prophylactic antibiotics or antiseptic agents would be administered to a woman by a healthcare provider.

We excluded studies on the clinical assessment, diagnosis or treatment of bacterial peripartum infections or their complications, and those reporting solely on the effectiveness, prevalence or extent of use of the specified interventions. We also did not consider other types of infection (such as viral or parasitic infections).

#### Information sources and search

We searched seven electronic databases for records dated from 1 January 1990 to 27 May 2022: MEDLINE (Ovid), EMBASE (Ovid), Emcare (Ovid), CINAHL (EbscoHost), Global Health (Ovid), Global Index Medicus, and Maternity and Infant Care (Ovid). The timeframe of 30 years was chosen to reflect contemporary maternity practice. Search terms were developed in consultation with an information specialist, and included search terms of synonyms for antibiotics, antiseptic agents, birth and prophylaxis (full search strategy in S2 Appendix). We also searched for relevant grey literature via OpenGrey (www.opengrey.eu), Agency for Healthcare Research and Quality (AHRQ; www.ahrq.gov), National Institute for Health and Clinical Excellence (NICE; www.nice.org.uk), and EThOs.

#### Study selection

Titles and abstracts of all search results were imported into Endnote and duplicates removed. We used Covidence for screening titles and abstracts and full texts [24]. Two review authors independently reviewed each title and abstract against the eligibility criteria, with potentially relevant articles included for full-text review. Full texts were retrieved and independently assessed for eligibility by two review authors. Disagreements at any stage were resolved by discussion or by involving a third reviewer. Where more than one paper reported the same study, the papers were collated to ensure the primary study is the unit of interest [21].

#### Assessing the methodological limitations of included studies

Critical appraisal of qualitative studies was conducted using an adaptation of the Critical Skills Appraisal Programme (CASP) tool including assessment of the following domains: study aims, methodology, design, recruitment, data collection, data analysis, reflexivity, ethical considerations, findings, and research contribution [25]. Critical appraisal of quantitative studies was conducted using the Newcastle-Ottawa scale for observational designs, adapted for cross-sectional studies, including the following domains: selection, comparability, outcome measures and analysis [26, 27]. No randomised trials or other quantitative study designs were eligible for inclusion. Given that synthesis was conducted separately for qualitative and quantitative data, separate critical appraisal assessments were conducted for each data type in mixed-methods studies. All methodological assessments were reviewed by a second study author, with disagreements resolved through discussion or consulting a third author. We did not exclude studies based on critical appraisal alone, however information about methodological limitations was used to assess our confidence in review findings.

#### Data extraction and synthesis

The following data were extracted from relevant studies: study characteristics; information on how the study was designed and conducted to inform assessment of methodological limitations; qualitative data including themes, findings and quotations; and quantitative data including data source, outcome measures, and results. Relevant qualitative and quantitative data were extracted separately. All extracted data were reviewed by a second reviewer and discrepancies were discussed until consensus was reached.

Synthesis was conducted separately for antibiotics and antiseptic agents given that the factors affecting their use may differ, and for each type of evidence (qualitative or quantitative). In the first stage of analysis, we used an inductive thematic synthesis approach for the qualitative data based on Thomas and Harden [28]. This included coding the relevant data and findings of all studies line-by-line using NVivo software, checking the text assigned to each code for consistency and any need for further division into sub-codes. A second reviewer checked the data within each code for consistency (RIZ). Higher-order analytical themes were developed through discussion between three reviewers from the codes to identify factors affecting use of the interventions. All codes were organised into a hierarchy grouping of related codes under these themes.

Given the considerable heterogeneity across the limited number of quantitative studies relevant to our research question (in terms of study aims, designs and outcomes reported) pooled meta-analysis was not performed, and quantitative results are reported narratively. Results from quantitative studies were mapped to the qualitative findings identified during the first stage of analysis. Together, these descriptive themes reflect findings from all included studies, regardless of methodology. To further explore how and why providers use prophylactic antibiotics, findings were mapped to a behaviour change framework based on Capability, Opportunity and Motivation as determinants of Behaviour (the COM-B model) [29]. This framework identifies three broad domains that must be addressed in order for behaviour change to occur–capability (a person’s psychological and physical capacity to perform a behaviour), opportunity (the social and physical factors that make a behaviour possible) and motivation (reflective beliefs and automatic responses that influence behaviour) [29].

#### Assessing confidence or certainty in the review findings

We used the GRADE-CERQual (Confidence in the Evidence from Reviews of Qualitative research) approach to assess our confidence in each qualitative finding, based on four key components [30]: methodological limitations of included studies [31], coherence of the review finding [32], adequacy of data [33], and relevance of included studies to the review question [34]. After assessing the degree of concerns (no or very minor, minor, moderate, or serious) regarding each of the four components, we made a judgement about our overall confidence in the evidence supporting the review finding (high, moderate, low, or very low) based on consensus among review authors [35]. In line with GRADE-CERQual guidance, all findings started at high confidence and were graded down if important concerns were raised. Given the available quantitative data could not be meta-analysed, the corresponding Newcastle-Ottawa quality rating of each study was reported for quantitative study findings.

#### Review author reflexivity

We maintained a reflexive stance throughout the stages of the review process, from study selection to data synthesis. At the outset of the review, our team considered that antibiotic and antiseptic use can be beneficial to prevent peripartum infections in some clinical situations, recognising that both interventions can be misused. Our team comes from multi-disciplinary backgrounds (medicine, midwifery, social sciences, public health), and progress was discussed regularly among the team and decisions made explored critically [21, 36].

## Results

**Fig 1:**
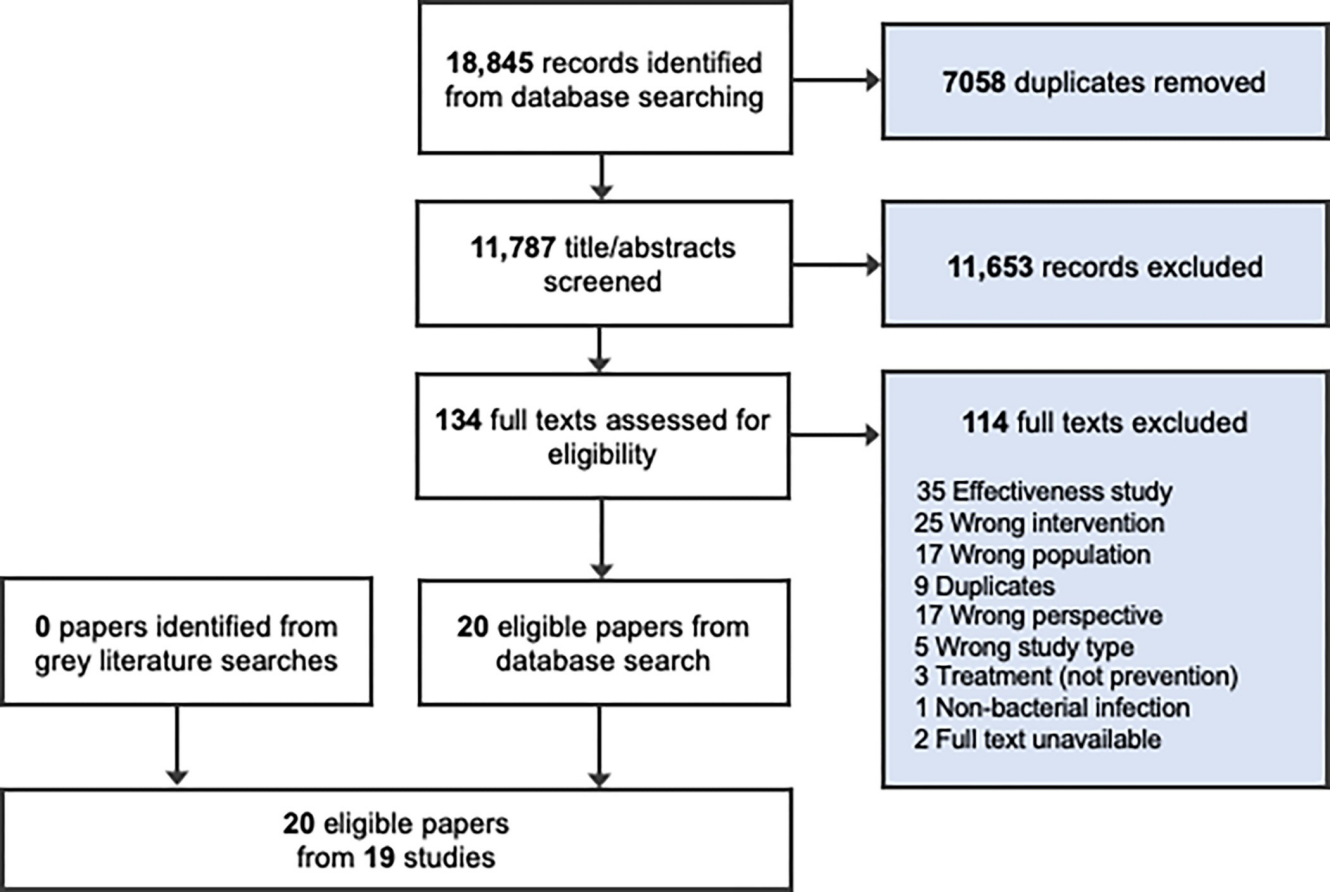
PRISMA flow diagram.

We identified 20 papers from 19 studies that fulfilled the inclusion criteria and are included in this synthesis (Fig 1).

A total of 5 qualitative, 12 quantitative and 2 mixed-methods studies were included (Table 1). Sixteen studies considered the use of prophylactic antibiotics (3 qualitative, 2 mixed methods, and 11 quantitative) [20, 37–52] and 2 considered the use of antiseptics (1 qualitative, 1 quantitative), and 1 considered the use of antibiotics and antiseptics (1 quantitative). Findings are reported separately for each intervention. Most studies explored the perspectives and experiences of health providers [20, 37–53], while 3 studies included relevant data from the perspectives of women [19, 54, 55]. Included studies were published between 1990 and 2020, and were conducted in 11 countries: Canada [43], Denmark [54], France [48], Ghana [49], Israel [41], Netherlands [42], Nigeria [38], and South Africa [46]; Thailand [20, 44], United Kingdom [19, 37, 45, 50], and United States of America (USA) [39, 40, 47, 51, 52]. All but 4 studies [20, 38, 44, 46, 49] were conducted in high-income countries.

**Table 1:** Characteristics of included studies.

Lead author and year	Intervention	Country (income level)	Methods	Data collection method(s)	Type and number of participants ^a^	Antimicrobial agent(s) if specified	Women characteristics
**Berrow 1997 [37]**	Antibiotics	England (High income)	Qualitative	Documentary analysis, observation, semi-structured interviews, and open-ended questionnaires	Unit staff of three obstetric units	Antibiotics (not otherwise specified)	All pregnant women
**Brisibe 2014 [38]**	Antibiotics and antiseptics	Nigeria (Lower middle income)	Quantitative	Structured questionnaire and observation	68 doctors and nurses	Antibiotic and antiseptic agents not specified	Women undergoing caesarean
**Edwards 2015 [39]**	Antibiotics	USA (High income)	Quantitative	Survey	273 members of the American College of Obstetricians and Gynecologists	Penicillin, ampicillin, cefazolin, clindamycin, vancomycin, and erythromycin	Women screened for GBS
**Everitt 1990 [40]**	Antibiotics	USA (High income)	Mixed methods	Audit, intervention trial with time-series analysis, interviews	In house officers on the obstetrics and gynaecology service (number not specified)	Cefazolin	Women undergoing caesarean
**Goldstick 2005 [41]**	Antibiotics	Israel (High income)	Quantitative	Telephone questionnaire	26 delivery unit directors and senior obstetricians	Antibiotics (not otherwise specified)	Women at risk of GBS
**Høgh-Poulsen (2021) [54]**	Antibiotics	Denmark (High income)	Qualitative	Semi-structured interviewes	14 pregnant women	Antibiotics (not otherwise specified)	Women considering or having a planned caesarean section, or scheduled for induction due to post-term
**Jakes 2020 [55]**	Antiseptics	UK (High income)	Quantitative	Questionnaire	20 women, 1 day following vaginal preparation	10% povidone-iodine solution. If allergic, chlorhexidine 2% aqueous solution	Women undergoing category II or III caesarean
**Kolkman 2017 [42]**	Antibiotics	Netherlands (High income)	Qualitative	FGDs and interviews	41 midwives, obstetricians, paediatricians, and microbiologists	Antibiotics (not otherwise specified)	Women at risk of GBS
**Konrad 2007 [43]**	Antibiotics	Canada (High income)	Quantitative	Population-based survey (interviews)	85 family physician and obstetrician practices	Antibiotics (not otherwise specified)	Women at risk of GBS
**Liabsuetrakul 2002[44] & 2003 [20]**	Antibiotics	Thailand (Upper middle income)	Mixed methods	Medical record review, questionnaire, and IDIs	50 obstetricians	Antibiotics (not otherwise specified)	Women undergoing caesarean
**Muthukumarappan 2000 [45]**	Antibiotics	UK (High income)	Quantitative	Case records review (audit), telephone interviews	An audit team comprising Clinical Governance Support Officer, a Consultant and Registrar Obstetrician and various labour ward medical and midwifery staff	Augmentin or Cefuroxime	Women undergoing caesarean
**Price 2018 [46]**	Antibiotics	South Africa (Upper middle income)	Quantitative	Questionnaires, FGDs	Doctors and maternity nurses—238 questionnaire respondents and two focus groups	Antibiotics (not otherwise specified)	Women at risk of GBS
**Raghunathan 2013 [47]**	Antibiotics	USA (High income)	Quantitative	Online survey	1052 anaesthetists	Antibiotics (not otherwise specified)	Women undergoing caesarean
**Rambourdin 2013 [48]**	Antibiotics	France (High income)	Quantitative	Postal survey	46 paediatricians	Antibiotics (not otherwise specified)	Women undergoing caesarean
**Sumankuuro 2018 [49]**	Antibiotics	Ghana (Lower middle income)	Qualitative	FGDs and IDIs	13 pharmacists, medical doctors, district directors of health services, midwives, community health and enrolled nurses	Antibiotics (not otherwise specified)	Pregnant women
**Tully 2002 [50]**	Antibiotics	UK (High income)	Quantitative	Questionnaire	2990 obstetricians	Antibiotics (not otherwise specified)	Women undergoing caesarean
**Watson 2019 [51]**	Antibiotics	USA (High income)	Quantitative	Online, self-administered survey	66 obstetricians and gynaecologists	Azithromycin	Women undergoing caesarean
**Watt 2001 [52]**	Antibiotics	USA (High income)	Quantitative	Survey questionnaire	702 members of the American College of Obstetricians and Gynecologists	Antibiotics (not otherwise specified)	Women at risk of GBS
**Weckesser 2019 [19]**	Antiseptics	England (High income)	Qualitative	FGDs and IDIs	21 women	Chlorhexidine	Women who had undergone caesarean within the preceding six months

FGD = focus group discussion; IDI = in-depth interview

^a^ where studies included multiple participant types, only those who provided eligible data for extraction in this review are mentioned

Detailed critical appraisals are available in S1 and S2 Tables. Of the 7 studies with qualitative data (including mixed-methods studies), we had no or very minor concerns about 4 studies [19, 20, 44, 49]. Two studies presented minor concerns (recruitment, reflexivity, and ethical approval not stated) [42, 54] and two studies presented serious concerns (research design, recruitment, reflexivity, ethical issues, data analysis and support for findings from the evidence) [37, 40]. Of the 16 studies with quantitative data (including mixed-methods), all used cross-sectional surveys. The quality of included studies (based on Newcastle-Ottawa score) ranges from good (6 studies) to satisfactory (7 studies) to unsatisfactory (2 studies), due to insufficient consideration of non-respondents; use of non-validated measurement tools; no adjustment for key potential confounders; and no statistical test used.

### Findings on use of prophylactic antibiotics to prevent peripartum infections

All relevant qualitative data from 6 studies reflected the perspectives of healthcare providers. Thirteen descriptive themes were identified, grouped under four second-order themes: (1) provider beliefs about benefits and harms; (2) provider perceptions of infection risk; (3) provider preferences regarding prophylactic antibiotic regimens and administration; and (4) other factors influencing provider decision-making on prophylactic antibiotic use. Only one study reported the perspectives from pregnant women, thus narratively described below [54]. Table 2 presents the summary of qualitative findings and GRADE-CERQual assessments. Findings from quantitative evidence were mapped to the qualitative findings and are presented in Table 3. The full GRADE-CERQual evidence profile is available at S3 Table.

**Table 2:** Summary of qualitative findings on perspectives and experiences of healthcare providers on use of peripartum prophylactic antibiotic.

Themes and summary of review finding	Contributing studies	GRADE-CERQual assessment
***Provider beliefs about benefits and harms of prophylactic antibiotic***		
Providers have mixed views on whether prophylactic antibiotics are effective and beneficial for preventing infection.	[20, 37, 44]	**Low confidence:** Minor concerns about methodological limitations. Serious concerns about relevance (upper-middle to high income countries from two regions) and adequacy (two studies).
Some physicians are more likely to use antibiotics for high-risk women undergoing caesarean section or following complications during the procedure, and less likely to prescribe for women undergoing elective caesarean section. Others use antibiotics routinely for all women undergoing caesarean section.	[20, 44]	**Very low confidence:** Serious concerns about relevance (upper-middle income country in one region) and adequacy (one study).
Some providers are concerned about unnecessary antibiotic use due to potential for unwanted side effects, overtreatment and medicalisation of birth, while others consider adverse reactions are low and outweighed by harm from infection.	[20, 42, 44]	**Low confidence:** Minor concerns regarding methodological limitations. Serious concerns about relevance (upper-middle to high income countries from two regions) and adequacy (two studies).
Providers have varying levels of concern about antimicrobial resistance—some prescribe less antibiotics for this reason, while others consider it is not a threat and have not changed their antibiotic prescription practice.	[20, 37, 42]	**Low confidence:** Moderate concerns about methodological limitations. Serious concerns about relevance (upper-middle income countries in two regions) and adequacy (three studies).
***Provider perceptions of infection risks***		
Some physicians are motivated by a fear of post-operative infection, and the risk of resulting blame and damage to their professional reputation. This can lead to a belief that erring on the side of overtreatment is preferable to undertreatment.	[20, 44]	**Very low confidence:** Serious concerns about relevance (upper-middle income country in one region) and adequacy (one study).
The risk of infection, and therefore the need for antibiotics, is considered by some providers to vary depending on local environmental factors.	[37, 44]	**Low confidence:** Moderate concerns about methodological limitations. Serious concerns about relevance (upper-middle to high income countries in two regions) and adequacy (two studies).
***Provider preferences regarding prophylactic antibiotic regimens and administration***		
Providers’ choice of a particular antibiotic agent is informed by whether it is recommended or common practice and perceptions of its effectiveness relative to other options.	[40, 44]	**Low confidence:** Moderate concerns about methodological limitations. Serious concerns about relevance (upper-middle to high income countries frm two settings) and adequacy (two studies).
Providers are influenced by locally recommended practices and personal experience in deciding how many doses to prescribe, with some believing multiple dose regimens are more effective.	[20, 44]	**Very low confidence:** Serious concerns about relevance (upper-middle income country in one region) and adequacy (one study).
Providers generally commence antibiotic administration after clamping the umbilical cord, with reasons including avoiding passing antimicrobial agents to the baby or in response to complications or potential contamination during surgery.	[40, 44]	**Low confidence:** Moderate concerns about methodological limitations. Serious concerns about relevance (upper-middle income country in two regions) and adequacy (two studies).
***Other factors influencing provider decision-making on prophylactic antibiotic use***		
Providers may have regard to the cost-effectiveness and affordability of antibiotics when deciding whether to prescribe and in choosing a particular antibiotic agent.	[20, 44, 49]	**Low confidence:** Serious concerns about relevance (middle income countries in two regions) and adequacy (two studies).
Some consider that the evidence regarding prophylactic antibiotics is not applicable to their local setting. They express a preference for evidence from local trials.	[20, 37]	**Low confidence:** Minor concerns about methodological limitations. Serious concerns about relevance (upper-middle income countries in two regions) and adequacy (two studies).
Providers obtain knowledge regarding appropriate antibiotic prescribing practices from varying sources. There are mixed views on the usefulness and uptake of guidelines. Some providers express preference for textbooks over journals.	[20, 44, 49]	**Low confidence:** Serious concerns about relevance (middle income countries in two regions) and adequacy (two studies).
Some providers antibiotic prescribing practices were highly influenced by professional norms and expectations, including pressure from colleagues and the observed practice of supervisors.	[20, 44]	**Very low confidence:** Serious concerns about relevance (upper-middle income country in one region) and adequacy (one study).

**Table 3:** Summary of findings from quantitative evidence on perspectives and experiences of women and healthcare providers on use of peripartum prophylactic antibiotics.

Theme	Summary of review finding	Contributing studies	Countries	Newcastle-Ottawa Quality Assessment
***Provider beliefs about benefits and harms of prophylactic antibiotic***				
*Provider beliefs about effectiveness of prophylactic antibiotics*	Many providers have a positive attitude toward administering prophylactic antibiotics as they believe these are effective for preventing infection. (Konrad 2007)	[20, 43]	Canada, Thailand	1 good study, 1 satisfactory study
*Provider beliefs about which women may benefit from prophylactic antibiotics *	Providers are more likely to administer prophylactic antibiotics for emergency CS than elective CS.	[50]	United Kingdom	1 satisfactory study
*Provider beliefs about side effects of prophylactic antibiotic use*	Some providers believe that the benefits of prophylactic antibiotics outweigh its risks, while others are concerned about the impact of antibiotic use on neonatal outcomes.	[43, 51]	Canada, United States	2 satisfactory studies
*Provider beliefs about antimicrobial resistance and whether this is important*	Provider attitudes towards broad-spectrum antibiotics can be negative due to concerns about drug resistance.	[20]	Thailand	1 good study
***Provider perceptions of infection risks***				
*Provider fears of maternal infection*	Providers’ prophylactic antibiotic prescribing practices are influenced by medico-legal considerations, including risk of lawsuits.	[41, 52]	Israel, United States	2 good studies
***Provider preferences regarding prophylactic antibiotic regimens and administration***				
*Provider attitudes towards using particular agents*	Many providers’ choice of antibiotic agent is based on the availability of drug stocks. Other factors include guidelines at time of residency, practice settings, and professional memberships.	[38, 39, 52]	Nigeria, United States (x2)	2 good studies, 1 satisfactory study
*Provider beliefs about number of doses of prophylactic antibiotics*	Some providers have unfavourable attitudes towards single-dose administration of prophylactic antibiotics as they consider it not to be cost-effective.	[20]	Thailand	1 good study
*Provider decisions about timing of administration of prophylactic antibiotics*	Preferences vary regarding the timing of prophylactic antibiotic administration, and this also depends on provider type (i.e., obstetrician, paediatrician, anaesthetist). For example, during caesarean section, some providers preferred pre-incision prophylaxis, and some intra-operative, including after cord-clamping. Factors underpinning timing choices include risk of maternal anaphylactic shock and the impact on newborns’ bacteriological samples and need for antibiotic therapy. For women at risk of GBS undergoing induction of labour, provider views on when to administer antibiotics similarly vary widely.	[20, 38, 39, 44, 47, 48, 51]	Nigeria, United States (x3), Thailand, France	3 good studies, 2 satisfactory studies, 1 unsatisfactory study
*Provider beliefs on who is responsible for prophylactic antibiotic administration*	Some providers believe administering antibiotics is an obstetric task and not the anaesthetists responsibility.	[45, 47]	United Kingdom, United States	1 good study, 1 satisfactory study
***Other factors influencing provider decision-making on prophylactic antibiotic use***				
*Provider beliefs about cost implications*	Some providers consider that drug costs are relevant in deciding antibiotic regimens, others believe that antibiotic use does not affect hospital costs.	[20, 43]	Canada, Thailand	1 good study, 1 satisfactory study
*Provider perceptions of the applicability of evidence to local settings*	Some providers are unaware of evidence regarding prophylactic antibiotics. Those who are aware still may not use antibiotics in practice due to perceived inadequacy of evidence, doubts about benefits, lack of training and absence of local guidelines or protocols regarding its use.	[38, 51]	Nigeria, United States	1 good study, 1 satisfactory study
*Influence of written reference materials (e*.*g*. *textbooks*, *journals*, *and guidelines)*	Published guidelines, regulations, scientific journals, textbooks, teaching curriculums, and hospital policy can influence providers’ prophylactic antibiotic use. Some providers consider guidelines are influential, important and would change their practice in response to updated policy. Some providers rank local hospital policy lower than journals and professional association publications.	[20, 39, 41, 52]	Israel, Thailand, United States (x2)	3 good studies, 1 satisfactory study
*Influence of professional norms and expectations*	Providers decisions regarding antibiotic prophylaxis are influenced to some degree by the views of others, including supervisors, specialists, senior and same-level colleagues.	[20, 38]	Nigeria, Thailand	2 good studies
***Strategies to influence prophylactic antibiotic use***				
*Provision of infection control training to providers*	Lack of training and knowledge is one factor underpinning providers’ non-compliance with prophylactic antibiotic administration recommendations.	[38, 46]	Nigeria, South Africa	1 good study, 1 satisfactory study
*Providers’ knowledge and compliance with guidelines and protocols*	Absence of local policy is a barrier to appropriate prophylactic antibiotic use, and implementing local guidelines, policy, and protocols can influence use. However, providers may not comply with guidelines due to lack of awareness or poor supervision.	[38, 39, 41]	United States, Israel, South Africa	2 good studies, 1 satisfactory study

*Provider beliefs about benefits and harms of prophylactic antibiotic use*. Qualitative research found providers had mixed views on whether prophylactic antibiotics are effective and beneficial for preventing infection (*low confidence*) [20, 37, 44]. Provider views varied regarding the indications for use. Some routinely used antibiotic prophylaxis only for women considered high-risk, such as women undergoing emergency caesarean section or if post-operative complications occurred. Others used prophylactic antibiotics routinely for all women undergoing caesarean section (*very low confidence*) [20, 44]. Some providers were concerned about unnecessary antibiotic use due to the potential for unwanted side effects, overtreatment and medicalisation of birth, while others considered the risk of adverse reactions to be low, and outweighed by the risk of harm due to infection (*low confidence*) [20, 42, 44]. Providers had varying levels of concern about antimicrobial resistance—some prescribe less antibiotics for this reason, while others did not consider it a threat and have not changed their prescribing practices (*low confidence*) [20, 37, 42].

Analysis of quantitative evidence similarly found that providers weighed various benefits and risks in deciding whether to use prophylactic antibiotics [20, 43, 51]. Many had a positive attitude toward administering prophylactic antibiotics [20, 43]. However, some reported that they were more likely to administer prophylactic antibiotics for emergency caesarean section than elective caesarean section [50]. Some providers in high-income countries (USA and Canada) believed that benefits of prophylactic antibiotics outweigh its risks, while some were concerned about the impact of antibiotic use on neonatal outcomes [43, 51].

*Provider perceptions of infection risk*. Qualitative evidence indicated that providers may be motivated by a fear of post-operative infection and the risk of resulting blame and damage to their professional reputation, leading to a belief that erring on the side of overtreatment is preferable (*very low confidence*) [20, 44]. The risk of infection, and therefore the need for antibiotic prophylaxis, was considered to vary depending on environmental factors, such as local infection rates and whether adequate infection control measures were in place at their facility (*low confidence*) [37, 44]. Surveys of providers in high-income countries (Israel and USA) found that sometimes concerns about medico-legal risk motivated decisions to adopt particular protocols and practices for antibiotic use, supporting the qualitative finding regarding fear of blame and reputational damage [41, 52].

*Provider preferences regarding prophylactic antibiotic regimens and administration*. Qualitative evidence found that providers had variable preferences regarding prophylactic antibiotic regimens. Their choice of antibiotic was affected by whether it was recommended or common practice, as well as perceptions of its effectiveness relative to other options (*low confidence*) [40, 44]. In deciding how many doses to prescribe, providers were influenced by locally recommended practices and personal experience. Some believed multiple dose regimens are more effective despite evidence of the effectiveness of single dose regimens (*very low confidence*) [20, 44]. Some providers reportedly commenced prophylactic antibiotic administration after clamping the umbilical cord for caesarean section. Reasons included to avoid passing antimicrobial agents to the baby, and to mitigate increased infection risk arising from complications or potential contamination during surgery (*low confidence*) [40, 44].

Quantitative evidence suggests that in practice many providers’ choice of an antibiotic is based on drug availability [38, 39, 52]. Quantitative studies indicated a variety of preferences for timing of antibiotic administration for caesarean section—some providers in France and the USA preferred pre-incision prophylaxis [47, 48, 51], while in Nigeria some preferred intra-operative administration [38], and in Thailand administration after umbilical cord-clamping [20, 44]. These preferences also varied by type of providers—paediatricians and anaesthetists were reported to prefer pre-incision prophylaxis [47, 48], yet obstetricians were reported to prefer administration after cord clamping [20]. Factors underpinning this decision included risk of maternal anaphylactic shock and the impact on newborns’ bacteriological samples and need for antibiotic therapy [48]. Some providers considered single-dose administration to be not cost-effective [20]. Providers also believed administrating antibiotics is an obstetric task and not an anaesthetist’s responsibility [45, 47], which demonstrates that lack of clarity on decision making responsibility may impact provider decision making.

*Other factors influencing provider decision-making on prophylactic antibiotic use*. Qualitative studies found that providers may consider cost-effectiveness for the health facility and affordability for the patient in making decisions about whether to use prophylactic antibiotics, and what agent to use (*low confidence*) [20, 44, 49]. Obstetricians and obstetric unit staff in questioned whether international effectiveness evidence regarding prophylactic antibiotics is applicable to their local setting, and expressed a preference for evidence from local trials (*low confidence*) [20, 37]. This complements the finding that infection risk is perceived to vary depending on the environment, informing providers’ perception of localised costs and benefits of antibiotics.

Providers reported their decision-making about prophylactic antibiotic use is informed by a range of written reference materials. There were mixed views on the usefulness and uptake of guidelines–for example, providers in Thailand expressed a preference for textbooks over journals (*low confidence*) [20, 44, 49]. Nurses in Ghana raised concerns that guidelines were not implemented in practice [49]. Thai obstetricians’ prescribing practices were highly influenced by professional norms and expectations, including pressure from colleagues and the observed practice of supervisors (*very low confidence*) [20, 44]. This was related to their fear of blame for adverse events but also reflected respect for supervisors’ knowledge and expertise.

Quantitative studies found providers were influenced by guidelines, regulations, journals, textbooks, teaching curriculums, and hospital policy [20, 39, 41, 52]. Despite guidelines and protocols existing at national or facility level, however, not all providers were aware of evidence regarding prophylactic antibiotic use. Those who were aware may not use antibiotics correctly in practice due to perceived inadequacy of evidence, doubts about benefits, lack of training, and absence of local guidelines or protocols regarding its use [38, 51]. Providers had mixed views on the usefulness and uptake of guidelines and policies [20, 39, 41, 52]. Similar to qualitative evidence, providers’ decisions regarding antibiotic prophylaxis are influenced to some degree by the views of others, including supervisors, specialists, senior and same-level colleagues. Providers consider the cost-effectiveness to some degree in administering antibiotics [20, 43].

### Factors influencing provider decisions to use prophylactic antibiotics at birth

**Fig 2:**
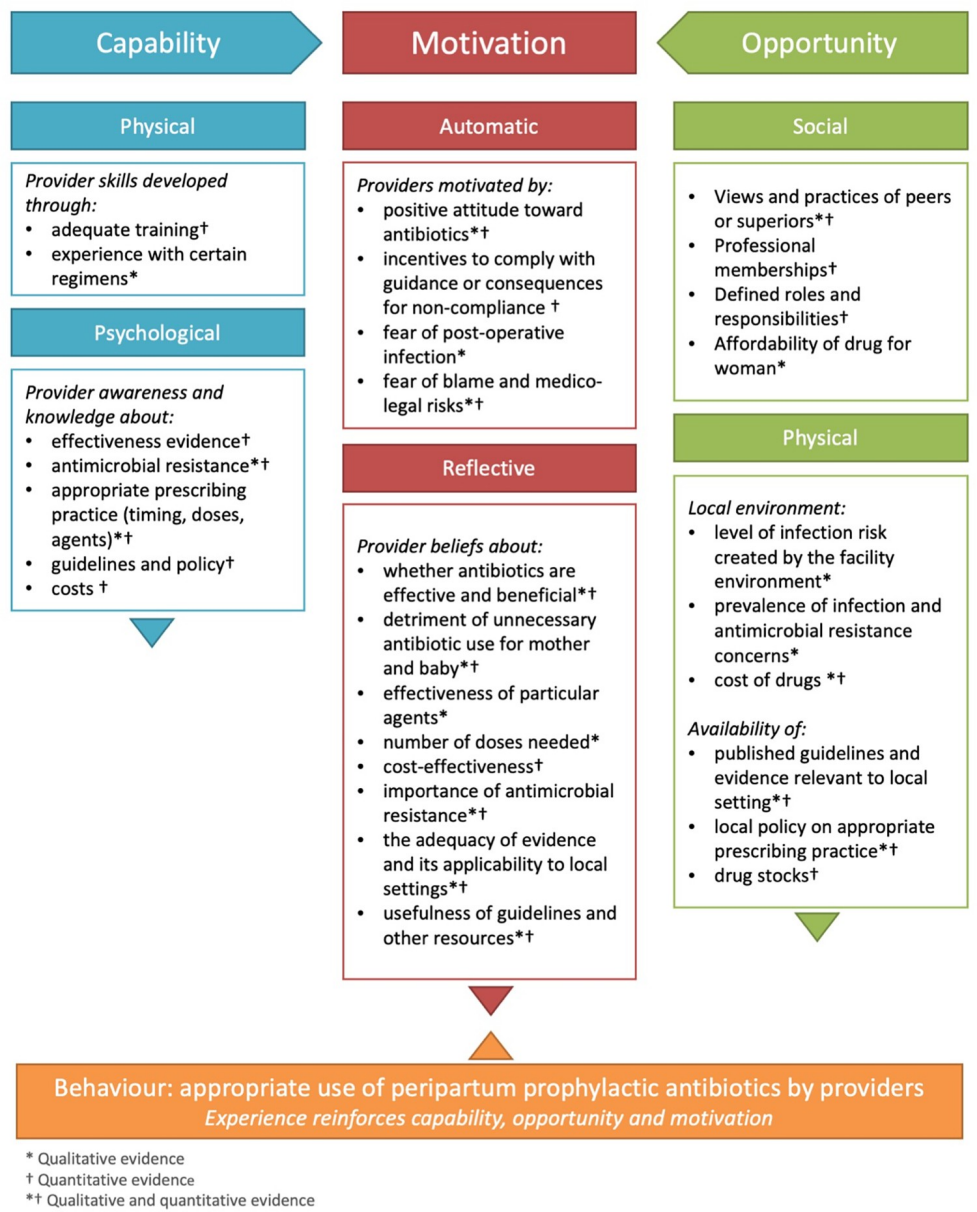
Factors affecting peripartum prophylactic antibiotic prescribing behaviour.

Findings from qualitative and quantitative evidence suggest providers’ decisions about whether and how to use prophylactic antibiotics at birth are complex and based on explicit or implicit consideration of a range of factors. We developed a framework of those factors affecting provider’s use of prophylactic antibiotics at birth using COM-B (Fig 2). We mapped factors under physical and psychological capability (Capability domain), physical and social opportunity (Opportunity domain), and how the interaction between these domains can influence provider motivation towards the behaviour of interest, i.e. appropriate use of peripartum prophylactic antibiotics. That is, when providers have improved awareness, skills and experience around correct prophylactic antibiotic use, we would expect positive changes to provider motivation in using antibiotics appropriately. Aligning social factors (such as peers, superiors and professional groups supportive of good prescribing practice) and physical factors (such as the clinical environment, as well as the availability of guidelines, policies and medicines) can also benefit motivation. These Capability and Opportunity domains can affect provider’s motivations, such as their attitudes, fears and beliefs around prophylactic antibiotic use.

*Women’s perceptions of antibiotics use during caesarean section*. Only one qualitative study reported the perspectives of Danish pregnant women on antibiotics during caesarean section [54]. Overall, women’s decisions on whether or when to receive antibiotics were related to concerns about the wellbeing of her baby and herself. Women had varied opinions on when they preferred to receive them—some women were concerned about possible impacts on their baby, the lack of scientific evidence around antibiotics, and they perceived most infections to not be serious. Some preferred receiving antibiotics after cord clamping, or not at all, to avoid risk to their baby. Other women, however, preferred receiving antibiotics pre-caesarean to minimise the risks of harm to themselves and their baby, feeling that they need to be well in order to take care of their baby. Women also described having limited knowledge about prophylactic antibiotics during caesarean section and desired more information. Many women reported trusting their healthcare providers’ judgement, even if it differed to their preference.

### Findings on use of antiseptic agents to prevent peripartum infections

Only three studies (one qualitative, two quantitative) considered use of antiseptic agents for vaginal cleansing and surgical skin preparation [19, 38, 55]. As too few studies were available for a meaningful synthesis, findings are reported narratively.

Weckesser et al explored women’s perspectives on caesarean section recovery and experiences of infection prevention in conjunction with the PREPS trial of vaginal cleansing with chlorhexidine immediately before caesarean section in the UK [19]. Prior to the trial, women expressed confusion about the purpose of vaginal cleansing with antiseptic agents. Once the rationale of reducing infection (endometritis) was explained, women perceived vaginal cleansing positively as an “upgrade” to standard practice. Women also considered that a detailed explanation of what the procedure involved in advance would likely be important to ensure its acceptability. One study from Nigeria considered the perspective of healthcare providers on antiseptic use for skin preparation before caesarean section [38]. A preference for specific antiseptic agents was due to health providers’ beliefs about its benefits, and some influence of guidelines. Health providers’ non-adherence to antiseptic use guidelines was reportedly due to lack of supervision, training, inadequate supplies, absence of facility-level policies or protocols to help implement guidelines, doubt about benefits, perceived lack of clinical evidence, and lack of examples or directives from senior colleagues. Changing practice to adhere to guidelines was attributed to experience with infection cases, medico-legal events, and provider’s change of beliefs about a specific regimen’s effectiveness. Jakes et al conducted an implementation study on vaginal preparation for women undergoing caesarean section, during which 20 women completed a questionnaire on their experience [55]. No women reported abnormal or discoloured vaginal discharge, vaginal irritation, pain, or concerns about discolouration of the baby’s scalp. Only one woman declined vaginal preparation during the implementation cycle.

## Discussion

This review identified factors affecting how providers make decisions to use prophylactic antibiotics around the time of birth, including their beliefs about benefits and harms, and context-specific infection risks. Providers have varying preferences for particular antibiotics and regimens, and may be influenced by their pre-existing beliefs on antimicrobial resistance, applicability of evidence, professional norms and expectations, and cost implications. There was no evidence on the views of women regarding peripartum antibiotic prophylaxis, including their acceptability of this intervention. Regarding antiseptic use at birth, the evidence was limited (four studies) hence meaningful synthesis was not possible and findings should be interpreted with caution.

Our findings on use of prophylactic antibiotics at birth are broadly aligned with previous reviews exploring physician antibiotic prescribing behaviour in non-obstetric disciplines. Our review found that provider beliefs about antibiotics affected use, and that providers are influenced by the behaviour of colleagues and supervisors. A 2009 systematic review of factors affecting use of perioperative prophylactic antibiotics in general surgery found practitioners were influenced by individual-level knowledge, attitudes and beliefs, team-level communication and responsibility, and institution-level promotion and monitoring activities [56]. Non-surgical antibiotic prescribing practices are also highly influenced by practices of fellow physicians, a factor also identified in our review [57–60], while non-surgical antibiotic use is influenced by patient expectations.

Multiple reviews have described that doctors may lack awareness or concern regarding the effect of their antibiotic prescribing behaviour on institution- or community-level antimicrobial resistance [57–59]; a phenomenon we likewise identified in relation to physicians’ decisions about peripartum antibiotic prophylaxis. Others have also reported that physicians may prescribe antibiotics due to fear of infection-related complications [57, 59]. Fear of repercussions could drive overuse while prescribing antibiotics provides comfort and alleviates perceived risk [61]. Finally, a related review of factors influencing adherence to guidelines on surgical antibiotic prophylaxis identified that provider training, personal experience and supervisors’ opinions may be stronger influencers on behaviour than clinical guidelines themselves [61].

We only found one study regarding women’s perceptions around peripartum antibiotic use, specifically during caesarean section [54]. In this study women’s decisions were influenced to the perceived safety of their baby, and many preferred not to take antibiotics if they were not necessary or medically indicated. Importantly, women reported having insufficient knowledge about antibiotics, and desired to learn more from their healthcare providers. It is nonetheless plausible that women giving birth may expect to receive antibiotics routinely, particularly in settings those where this practice is widespread.

There were limited data from low- and middle-income countries–only four studies were conducted in these settings. Overall, similar factors were observed across settings in relation to antibiotic use, which included pre-existing beliefs around benefits and harms, preferences, costs, and perceived of lack of guidelines or absence of local policy [20, 38, 39, 44, 47, 48, 51]. Minor differences, however, were observed. For example, lack of infection control training and knowledge were commonly reported in studies in low- and middle-income countries [38, 46], which might reflect broader or more systemic challenges to delivering maternity services [62]. Provider decisions around antibiotic prophylaxis use were also influenced by the view of senior colleagues such as supervisors [20, 38], suggesting that mentoring or engaging local champions from a senior staff level may be effective strategies to improve appropriate antibiotic use [63]. Regardless, it is clear that more studies are needed to understand factors affecting use of these interventions in limited-resource settings.

### Strengths and limitations

This is the first systematic review of currently available evidence on how women, partners, families and providers perceive and experience the use of antibiotic antiseptic agents for infection prevention at birth. Strengths of this review include a comprehensive search strategy, adherence to a pre-specified review protocol (including duplicate screening, extraction, critical appraisal and GRADE-CERQual assessments), as well as combining evidence from qualitative and quantitative data. However, the modest number of eligible studies limited our ability to draw strong conclusions. Furthermore, some studies had serious limitations regarding adequacy of evidence and relevance to global settings, resulting in low to very low confidence assessments. While quantitative evidence broadly supported the qualitative findings, the overall evidence base remains relatively limited and further research is required.

### Implications for practice, policy and research

In order to prevent death and disease due to peripartum infections, evidence-based guidelines are needed to optimise the use of prophylactic antibiotics and antiseptics around the time of childbirth. Consideration of factors we identified, like provider capability (such as their skills, experience and knowledge), their motivations and their clinical environments, are needed for to optimise strategies to improve prophylactic antibiotic use. These findings are useful in developing evidence-based guidelines, particularly in understanding stakeholder’s views, acceptability, feasibility and implementability of an intervention [33, 35]. For example, findings from this review have informed forthcoming updates of living WHO recommendations related to peripartum antibiotic and antiseptic use [64].

However, this review emphasises that our understanding of how women, their partners and families perceive use of antimicrobial agents in the peripartum period is limited. This gap should be addressed to ensure that women’s voices are included in how maternity care is delivered. Additional research is also needed to better understand how providers balance consideration of infection risk, the side effects of antibiotic use, and antimicrobial resistance as both a patient-specific and public health concern. There is a need for greater understanding of providers’ attitudes towards guidelines on antibiotic use, and barriers to guideline implementation in limited-resource settings, noting that most studies were from high-resource settings. With only three studies identified on antiseptic agents, further research on this intervention is also a priority. An improved evidence base will provide researchers and policymakers with further insights regarding why antibiotics and antiseptics may be misused in some settings, and inform the development of more effective implementation strategies to address these issues.

## Conclusions

This review identified a range of factors affecting how providers prescribe prophylactic antibiotics around the time of birth, which may lead to prescribing practices that are not in line with clinical guidelines. The limited available evidence base highlights the need for additional research, particularly regarding women’s perspectives on both antibiotic and antiseptic use, as well as factors affecting their use on low- and- middle income countries. Improving adherence to recommended practice will likely require multifaceted interventions that are adapted to address local contexts.

## Supporting information

S1 Appendix PRISMA checklist. (DOCX)

S2 Appendix Search strategies. (DOCX)

S1 Table CASP assessments of qualitative and mixed methods studies. (DOCX)

S2 Table Newcastle-Ottawa scale assessments of studies with quantitative methods. (DOCX)

S3 Table GRADE-CERQual evidence profile. (DOCX)
