# Supplementary material for: Factors affecting the use of antibiotics and antiseptics to prevent maternal infection at birth: A global mixed-methods systematic review
Source: PLoS One. 2022 Sep 1;17(9):e0272982. doi: 10.1371/journal.pone.0272982 (PMC9436089; doi:10.1371/journal.pone.0272982)
Supplement: S2 Appendix — (DOCX) [file pone.0272982.s002.docx]

# S2 Appendix. Search strategies

## Medline database search

Database(s): Ovid MEDLINE(R) and Epub Ahead of Print, In-Process & Other Non-Indexed Citations and Daily 1946 to 27 May 2022

| **#** | **Searches** |
| --- | --- |
| 1 | Obstetrics/ or "Obstetrics and Gynecology Department, Hospital"/ or obstetric surgical procedures/ or delivery, obstetric/ or cesarean section/ or cesarean section, repeat/ or episiotomy/ or extraction, obstetrical/ or vacuum extraction, obstetrical/ or labor, induced/ or vaginal birth after cesarean/ or labor, obstetric/ or labor onset/ or labor stage, first/ or labor stage, second/ or labor stage, third/ or labor presentation/ or "trial of labor"/ or obstetric labor complications/ or obstetric labor, premature/ or premature birth/ or parturition/ or birth setting/ or home childbirth/ or natural childbirth/ or term birth/ or birthing centers/ or delivery rooms/ or hospitals, maternity/ or Obstetric Nursing/ or Doulas/ or Midwifery/ or Nurse Midwives/ or Perinatal Care/ or Peripartum Period/ or Postpartum Period/ |
| 2 | Antibiotic Prophylaxis/ |
| 3 | Endometritis/pc [Prevention & Control] |
| 4 | exp infections/pc or exp sepsis/pc |
| 5 | Bacterial Infections/pc [Prevention & Control] |
| 6 | infection control/ or antisepsis/ or asepsis/ |
| 7 | 2 or 3 or 4 or 5 or 6 |
| 8 | 1 and 7 |
| 9 | exp anti-infective agents/ or exp anti-bacterial agents/ |
| 10 | infection control/ or (infect* adj3 prevent*).mp. |
| 11 | 1 and 9 and 10 |
| 12 | 8 or 11 |
| 13 | exp animals/ not humans.sh. |
| 14 | 12 not 13 |
| 15 | limit 14 to (case reports or comment or editorial or historical article or letter or news) |
| 16 | 14 not 15 |
| 17 | (obstetric* or childbirth* or birth*2 or intrapartum or intra-partum or antepartum or ante-partum or perinatal or peri-natal or peripartum or peri-partum or parturition or puerperal or maternity or midwives or midwifery or birthing or childbearing or cesar#an* or caesar#an* or c-section* or episiotom*).mp.  Alternative:  (childbirth* or or intrapartum or intra-partum or antepartum or ante-partum or perinatal or peri-natal or peripartum or peri-partum or parturition or puerperal or midwives or birthing or childbearing or cesar#an* or caesar#an* or c-section* or episiotom*).mp. |
| 18 | ((vagina* or placenta* or obstetric* or operative or procedur*2 or breech or pre-term or preterm or term or instrumental) adj3 deliver*).mp. |
| 19 | ((delivery or labor or labour) adj5 (pregnan* or neonatal or neo-natal or postnatal or post-natal or postpartum or post-partum or antenatal or ante-natal or antepartum or ante-partum or newborn or matern* or obstetric* or induction or inductive)).mp. |
| 20 | 17 or 18 or 19 |
| 21 | (antibiotic* or anti-biotic* or antibacterial* or anti-bacterial* or amikacin* or Aminoglycoside* or aminopenicillin* or amoxicillin* or Amphenicol* or ampicillin* or ampictam* or augmentin* or azithromycin* or aztreonam* or benzathine* or Benzylpenicillin* or beta-lactam* or carbapenem* or carbenicillin* or cefaclor* or cefadroxil* or cefamandole* or cefazolin* or cefcapene* or cefclidine* or cefdaloxime* or cefditoren* or Cefepime* or cefetamet* or cefixime* or cefluprenam* or cefmenoxime* or cefodizime* or cefonicid* or cefoperazone* or ceforanide* or Cefotaxime* or cefotetan* or cefotiam* or Cefotoxime* or Cefoxitin* or cefozopran* or cefpimizole* or cefpirome* or cefpodoxime* or cefprozil* or cefquinome* or ceftazidime* or ceftibuten* or ceftizoxime* or Ceftolozane* or ceftriaxone* or cefuroxime* or cephalexin* or cephalosporin* or Cephalothin* or cephamycin* or cephapirin* or cephradine* or Chloramphenicol* or Ciprofloxacin* or clarithromycin* or clavamox* or clavulanate* or clavulanic acid* or clinafloxacin* or clindamicin* or clindamycin* or cloxacillin* or co-amoxyclav* or dicloxacillin* or doxycycline* or ertapenem* or Erythromycin* or Fluoroquinolone* or gatifloxacin* or gentamicin* or imipenem* or kanamycin* or levofloxacin* or Lincomycin* or Lincosamide* or lomefloxacin* or Macrolide* or maxifloxacin* or meropenem* or methicillin* or Metronidazole* or mezlocillin* or minocycline* or nafcillin* or Nitroimidazole* or norfloxacin* or ofloxacin* or oxacillin* or penicillin* or piperacillin* or procaine* or sparfloxacin* or Streptomycin* or sulbactam* or Tetracycline* or ticarcillin* or timentin* or tinidazol* or trovafloxacin* or tyclav* or unasyn*).mp. |
| 22 | (antiseptic* or anti-septic* or vaginal cleans* or skin prep* or saline wash or benzalkonium chloride* or carbolic acid spray* or cetrimide* or chlorhexidine* or gluconate* or hexachlorophene* or iodine* or iodophor* or isopropyl* or mercuric compound* or parachlorometaxylenol* or povidone-iodine* or triclosan* or vaginal metronidazole*).mp. |
| 23 | 21 or 22 |
| 24 | ((sepsis or infection*) adj6 (prophylaxis or prophylac* or prevent*)).mp. |
| 25 | 20 and 23 and 24 |
| 26 | exp animals/ not humans.sh. |
| 27 | 25 not 26 |
| 28 | limit 27 to (case reports or comment or editorial or historical article or letter or news) |
| 29 | 27 not 28 |
| 30 | Puerperal Infection/pc [Prevention & Control] |
| 31 | Puerperal Infection/ |
| 32 | Antibiotic Prophylaxis/ or exp infections/pc or exp sepsis/pc or Bacterial Infections/pc or infection control/ or antisepsis/ or asepsis/ |
| 33 | 31 and 32 |
| 34 | 30 or 33 |
| 35 | exp animals/ not humans.sh. |
| 36 | 34 not 35 |
| 37 | limit 36 to (case reports or comment or editorial or historical article or letter or news) |
| 38 | 36 not 37 |
| 39 | 16 or 29 or 38 |
| 40 | limit 39 to yr="1990 -Current" |

## Embase database search

Database(s):  Embase Classic+Embase 1947 to 27 May 2022

| **#** | **Searches** |
| --- | --- |
| 1 | birth/ or childbirth/ or delivery room/ or obstetrician/ or obstetrics/ or obstetric anesthesia/ or obstetric analgesia/ or obstetric care/ or midwife/ or nurse midwife/ or doula/ or nurse midwifery/ or intrapartum care/ or obstetrical nursing/ or maternal care/ or maternity ward/ or perinatal care/ or perinatal period/ or puerperium/ or postnatal care/ or obstetric procedure/ or obstetric delivery/ or fetal version/ or home delivery/ or instrumental delivery/ or breech extraction/ or cesarean section/ or forceps delivery/ or vacuum extraction/ or obstetric operation/ or episiotomy/ or placental delivery/ or vaginal birth after cesarean/ or vaginal delivery/ or water birth/ or birth setting/ or natural childbirth/ or labor induction/ or labor management/ or labor support/ or labor/ or labor stage/ or labor onset/ or labor stage 1/ or labor stage 2/ or labor stage 3/ or trial of labor/ or term birth/ or labor complication/ |
| 2 | Antibiotic Prophylaxis/ |
| 3 | Endometritis/pc [Prevention & Control] |
| 4 | exp infections/pc or exp sepsis/pc |
| 5 | Bacterial Infections/pc [Prevention & Control] |
| 6 | infection control/ or antisepsis/ or asepsis/ |
| 7 | 2 or 3 or 4 or 5 or 6 |
| 8 | 1 and 7 |
| 9 | exp anti-infective agents/ |
| 10 | infection control/ or (infect* adj3 prevent*).mp. |
| 11 | 1 and 9 and 10 |
| 12 | 8 or 11 |
| 13 | (exp animal/ or exp invertebrate/ or animal.hw. or nonhuman/) not exp human/ |
| 14 | 12 not 13 |
| 15 | limit 14 to (conference abstract or editorial or erratum or letter or note) |
| 16 | 14 not 15 not case report*.mp. |
| 17 | (obstetric* or childbirth* or birth*2 or intrapartum or intra-partum or antepartum or ante-partum or perinatal or peri-natal or peripartum or peri-partum or parturition or puerperal or maternity or midwives or midwifery or birthing or childbearing or cesar#an* or caesar#an* or c-section* or episiotom*).mp. |
| 18 | ((vagina* or placenta* or obstetric* or operative or procedur*2 or breech or pre-term or preterm or term or instrumental) adj3 deliver*).mp. |
| 19 | ((delivery or labor or labour) adj5 (pregnan* or neonatal or neo-natal or postnatal or post-natal or postpartum or post-partum or antenatal or ante-natal or antepartum or ante-partum or newborn or matern* or obstetric* or induction or inductive)).mp. |
| 20 | 17 or 18 or 19 |
| 21 | (antibiotic* or anti-biotic* or antibacterial* or anti-bacterial* or amikacin* or Aminoglycoside* or aminopenicillin* or amoxicillin* or Amphenicol* or ampicillin* or ampictam* or augmentin* or azithromycin* or aztreonam* or benzathine* or Benzylpenicillin* or beta-lactam* or carbapenem* or carbenicillin* or cefaclor* or cefadroxil* or cefamandole* or cefazolin* or cefcapene* or cefclidine* or cefdaloxime* or cefditoren* or Cefepime* or cefetamet* or cefixime* or cefluprenam* or cefmenoxime* or cefodizime* or cefonicid* or cefoperazone* or ceforanide* or Cefotaxime* or cefotetan* or cefotiam* or Cefotoxime* or Cefoxitin* or cefozopran* or cefpimizole* or cefpirome* or cefpodoxime* or cefprozil* or cefquinome* or ceftazidime* or ceftibuten* or ceftizoxime* or Ceftolozane* or ceftriaxone* or cefuroxime* or cephalexin* or cephalosporin* or Cephalothin* or cephamycin* or cephapirin* or cephradine* or Chloramphenicol* or Ciprofloxacin* or clarithromycin* or clavamox* or clavulanate* or clavulanic acid* or clinafloxacin* or clindamicin* or clindamycin* or cloxacillin* or co-amoxyclav* or dicloxacillin* or doxycycline* or ertapenem* or Erythromycin* or Fluoroquinolone* or gatifloxacin* or gentamicin* or imipenem* or kanamycin* or levofloxacin* or Lincomycin* or Lincosamide* or lomefloxacin* or Macrolide* or maxifloxacin* or meropenem* or methicillin* or Metronidazole* or mezlocillin* or minocycline* or nafcillin* or Nitroimidazole* or norfloxacin* or ofloxacin* or oxacillin* or penicillin* or piperacillin* or procaine* or sparfloxacin* or Streptomycin* or sulbactam* or Tetracycline* or ticarcillin* or timentin* or tinidazol* or trovafloxacin* or tyclav* or unasyn*).mp. |
| 22 | (antiseptic* or anti-septic* or vaginal cleans* or skin prep* or saline wash or benzalkonium chloride* or carbolic acid spray* or cetrimide* or chlorhexidine* or gluconate* or hexachlorophene* or iodine* or iodophor* or isopropyl* or mercuric compound* or parachlorometaxylenol* or povidone-iodine* or triclosan* or vaginal metronidazole*).mp. |
| 23 | 21 or 22 |
| 24 | ((sepsis or infection*) adj6 (prophylaxis or prophylac* or prevent*)).mp. |
| 25 | 20 and 23 and 24 |
| 26 | (exp animal/ or exp invertebrate/ or animal.hw. or nonhuman/) not exp human/ |
| 27 | 25 not 26 |
| 28 | limit 27 to (conference abstract or editorial or erratum or letter or note) |
| 29 | 27 not 28 not case report*.mp. |
| 30 | Puerperal Infection/pc [Prevention & Control] |
| 31 | Puerperal Infection/ |
| 32 | Antibiotic Prophylaxis/ or exp infections/pc or exp sepsis/pc or Bacterial Infections/pc or infection control/ or antisepsis/ or asepsis/ |
| 33 | 31 and 32 |
| 34 | 30 or 33 |
| 35 | (exp animal/ or exp invertebrate/ or animal.hw. or nonhuman/) not exp human/ |
| 36 | 34 not 35 |
| 37 | limit 36 to (conference abstract or editorial or erratum or letter or note) |
| 38 | 36 not 37 not case report*.mp |

## Emcare database search

Database(s): Ovid Emcare 1995 to 27 May 2022

| **#** | **Searches** |
| --- | --- |
| 1 | birth/ or childbirth/ or delivery room/ or obstetrician/ or obstetrics/ or obstetric anesthesia/ or obstetric analgesia/ or obstetric care/ or midwife/ or nurse midwife/ or doula/ or nurse midwifery/ or intrapartum care/ or obstetrical nursing/ or maternal care/ or maternity ward/ or perinatal care/ or perinatal period/ or puerperium/ or postnatal care/ or obstetric procedure/ or obstetric delivery/ or fetal version/ or home delivery/ or instrumental delivery/ or breech extraction/ or cesarean section/ or forceps delivery/ or vacuum extraction/ or obstetric operation/ or episiotomy/ or placental delivery/ or vaginal birth after cesarean/ or vaginal delivery/ or water birth/ or birth setting/ or natural childbirth/ or labor induction/ or labor management/ or labor support/ or labor/ or labor stage/ or labor onset/ or labor stage 1/ or labor stage 2/ or labor stage 3/ or trial of labor/ or term birth/ or labor complication/ |
| 2 | Antibiotic Prophylaxis/ |
| 3 | Endometritis/ or infection/ or sepsis/ or bacterial infection/ or perinatal infection/ or surgical infection/ |
| 4 | infection prevention/ or infection control/ or antisepsis/ or asepsis/ or (infect* adj3 prevent*) or (infect* adj3 prophylaxis*) |
| 5 | infection prevention/ or antisepsis/ or asepsis/ |
| 6 | 3 and 4 |
| 7 | 2 or 5 or 6 |
| 8 | 1 and 7 |
| 9 | exp anti-infective agents/ |
| 10 | infection control/ or (infect* adj3 prevent*).mp. |
| 11 | 1 and 9 and 10 |
| 12 | 8 or 11 |
| 13 | (exp animal/ or exp invertebrate/ or animal.hw. or nonhuman/) not exp human/ |
| 14 | 12 not 13 |
| 15 | limit 14 to (conference abstract or editorial or erratum or letter or note) |
| 16 | 14 not 15 not case report*.mp. |
| 17 | (obstetric* or childbirth* or birth*2 or intrapartum or intra-partum or antepartum or ante-partum or perinatal or peri-natal or peripartum or peri-partum or parturition or puerperal or maternity or midwives or midwifery or birthing or childbearing or cesar#an* or caesar#an* or c-section* or episiotom*).mp. |
| 18 | ((vagina* or placenta* or obstetric* or operative or procedur*2 or breech or pre-term or preterm or term or instrumental) adj3 deliver*).mp. |
| 19 | ((delivery or labor or labour) adj5 (pregnan* or neonatal or neo-natal or postnatal or post-natal or postpartum or post-partum or antenatal or ante-natal or antepartum or ante-partum or newborn or matern* or obstetric* or induction or inductive)).mp. |
| 20 | 17 or 18 or 19 |
| 21 | (antibiotic* or anti-biotic* or antibacterial* or anti-bacterial* or amikacin* or Aminoglycoside* or aminopenicillin* or amoxicillin* or Amphenicol* or ampicillin* or ampictam* or augmentin* or azithromycin* or aztreonam* or benzathine* or Benzylpenicillin* or beta-lactam* or carbapenem* or carbenicillin* or cefaclor* or cefadroxil* or cefamandole* or cefazolin* or cefcapene* or cefclidine* or cefdaloxime* or cefditoren* or Cefepime* or cefetamet* or cefixime* or cefluprenam* or cefmenoxime* or cefodizime* or cefonicid* or cefoperazone* or ceforanide* or Cefotaxime* or cefotetan* or cefotiam* or Cefotoxime* or Cefoxitin* or cefozopran* or cefpimizole* or cefpirome* or cefpodoxime* or cefprozil* or cefquinome* or ceftazidime* or ceftibuten* or ceftizoxime* or Ceftolozane* or ceftriaxone* or cefuroxime* or cephalexin* or cephalosporin* or Cephalothin* or cephamycin* or cephapirin* or cephradine* or Chloramphenicol* or Ciprofloxacin* or clarithromycin* or clavamox* or clavulanate* or clavulanic acid* or clinafloxacin* or clindamicin* or clindamycin* or cloxacillin* or co-amoxyclav* or dicloxacillin* or doxycycline* or ertapenem* or Erythromycin* or Fluoroquinolone* or gatifloxacin* or gentamicin* or imipenem* or kanamycin* or levofloxacin* or Lincomycin* or Lincosamide* or lomefloxacin* or Macrolide* or maxifloxacin* or meropenem* or methicillin* or Metronidazole* or mezlocillin* or minocycline* or nafcillin* or Nitroimidazole* or norfloxacin* or ofloxacin* or oxacillin* or penicillin* or piperacillin* or procaine* or sparfloxacin* or Streptomycin* or sulbactam* or Tetracycline* or ticarcillin* or timentin* or tinidazol* or trovafloxacin* or tyclav* or unasyn*).mp. |
| 22 | (antiseptic* or anti-septic* or vaginal cleans* or skin prep* or saline wash or benzalkonium chloride* or carbolic acid spray* or cetrimide* or chlorhexidine* or gluconate* or hexachlorophene* or iodine* or iodophor* or isopropyl* or mercuric compound* or parachlorometaxylenol* or povidone-iodine* or triclosan* or vaginal metronidazole*).mp. |
| 23 | 21 or 22 |
| 24 | ((sepsis or infection*) adj6 (prophylaxis or prophylac* or prevent*)).mp. |
| 25 | 20 and 23 and 24 |
| 26 | (exp animal/ or exp invertebrate/ or animal.hw. or nonhuman/) not exp human/ |
| 27 | 25 not 26 |
| 28 | limit 27 to (conference abstract or editorial or erratum or letter or note) |
| 29 | 27 not 28 not case report*.mp. |
| 30 | Puerperal Infection/ |
| 31 | prophylaxis or prophylac* or prevent*.mp |
| 32 | Antibiotic Prophylaxis/ or exp infection prevention/ or exp infection control/ or  Bacterial Infections/ or antisepsis/ or asepsis/.mp |
| 33 | 31 or 32 |
| 32 | 30 and 33 |
| 35 | (exp animal/ or exp invertebrate/ or animal.hw. or nonhuman/) not exp human/ |
| 36 | 34 not 35 |
| 37 | limit 36 to (conference abstract or editorial or erratum or letter or note) |
| 38 | 36 not 37 not case report*.mp |
| 39 | 16 or 29 or 38 |
| 40 | limit 39 to yr="1990 -Current" |

## Global Health database search

Database(s): Global Health 1910 to 27 May 2022

| **#** | **Searches** |
| --- | --- |
| 1 | Birth/ or childbirth/ or caesarean section/ or parturition/ or parturition complications/ or obstetrics/ or exp midwives/ or puerperium/ or Postpartum interval/ or postpartum period/ or pregnancy complications/ or maternity services/ or Puerperal disorders/ |
| 2 | exp antibiotics/ |
| 3 | Endometritis/ or infection/ or sepsis/ or Surgical site infections/ or preoperative care/ |
| 4 | infection control/ or (infect* adj3 prevent*) or (infect* adj3 prophylaxis*) or chemoprophylaxis/ or prophylaxis/ |
| 5 | 2 and 4 |
| 6 | 3 and 4 |
| 7 | 5 or 6 |
| 8 | 1 and 7 |
| 9 | antiinfective agents/ or exp antibacterial agents/ or exp antiseptics/ or exp dermatological agents/ |
| 10 | infection control/ or (infect* adj6 prevent*).mp. |
| 11 | 1 and 9 and 10 |
| 12 | 8 or 11 |
| 13 | (animals/ or laboratory animals/ or domestic animals/ or animal.hw.) not exp Hominidae/ |
| 14 | 12 not 13 |
| 15 | limit 14 to (conference paper or conference proceedings or correspondence or editorial) |
| 16 | 14 not 15 not case reports/ |
| 17 | (obstetric* or childbirth* or birth*2 or intrapartum or intra-partum or antepartum or ante-partum or perinatal or peri-natal or peripartum or peri-partum or parturition or puerperal or maternity or midwives or midwifery or birthing or childbearing or cesar#an* or caesar#an* or c-section* or episiotom*).mp. |
| 18 | ((vagina* or placenta* or obstetric* or operative or procedur*2 or breech or pre-term or preterm or term or instrumental) adj3 deliver*).mp. |
| 19 | ((delivery or labor or labour) adj5 (pregnan* or neonatal or neo-natal or postnatal or post-natal or postpartum or post-partum or antenatal or ante-natal or antepartum or ante-partum or newborn or matern* or obstetric* or induction or inductive)).mp. |
| 20 | 17 or 18 or 19 |
| 21 | (antibiotic* or anti-biotic* or antibacterial* or anti-bacterial* or amikacin* or Aminoglycoside* or aminopenicillin* or amoxicillin* or Amphenicol* or ampicillin* or ampictam* or augmentin* or azithromycin* or aztreonam* or benzathine* or Benzylpenicillin* or beta-lactam* or carbapenem* or carbenicillin* or cefaclor* or cefadroxil* or cefamandole* or cefazolin* or cefcapene* or cefclidine* or cefdaloxime* or cefditoren* or Cefepime* or cefetamet* or cefixime* or cefluprenam* or cefmenoxime* or cefodizime* or cefonicid* or cefoperazone* or ceforanide* or Cefotaxime* or cefotetan* or cefotiam* or Cefotoxime* or Cefoxitin* or cefozopran* or cefpimizole* or cefpirome* or cefpodoxime* or cefprozil* or cefquinome* or ceftazidime* or ceftibuten* or ceftizoxime* or Ceftolozane* or ceftriaxone* or cefuroxime* or cephalexin* or cephalosporin* or Cephalothin* or cephamycin* or cephapirin* or cephradine* or Chloramphenicol* or Ciprofloxacin* or clarithromycin* or clavamox* or clavulanate* or clavulanic acid* or clinafloxacin* or clindamicin* or clindamycin* or cloxacillin* or co-amoxyclav* or dicloxacillin* or doxycycline* or ertapenem* or Erythromycin* or Fluoroquinolone* or gatifloxacin* or gentamicin* or imipenem* or kanamycin* or levofloxacin* or Lincomycin* or Lincosamide* or lomefloxacin* or Macrolide* or maxifloxacin* or meropenem* or methicillin* or Metronidazole* or mezlocillin* or minocycline* or nafcillin* or Nitroimidazole* or norfloxacin* or ofloxacin* or oxacillin* or penicillin* or piperacillin* or procaine* or sparfloxacin* or Streptomycin* or sulbactam* or Tetracycline* or ticarcillin* or timentin* or tinidazol* or trovafloxacin* or tyclav* or unasyn*).mp. |
| 22 | (antiseptic* or anti-septic* or vaginal cleans* or skin prep* or saline wash or benzalkonium chloride* or carbolic acid spray* or cetrimide* or chlorhexidine* or gluconate* or hexachlorophene* or iodine* or iodophor* or isopropyl* or mercuric compound* or parachlorometaxylenol* or povidone-iodine* or triclosan* or vaginal metronidazole*).mp. |
| 23 | 21 or 22 |
| 24 | ((sepsis or infection*) adj6 (prophylaxis or prophylac* or prevent*)).mp. |
| 25 | 20 and 23 and 24 |
| 26 | (animals/ or laboratory animals/ or domestic animals/ or animal.hw.) not exp Hominidae/ |
| 27 | 25 not 26 |
| 28 | limit 27 to (conference paper or conference proceedings or correspondence or editorial) |
| 29 | 27 not 28 not case reports/ |
| 30 | 16 or 29 |
| 31 | limit 30 to yr="1990 -Current" |

## Global Index Medicus database search

| (tw:((obstetric* or childbirth* or birth* or intrapartum or intra-partum or antepartum or ante-partum or perinatal or peri-natal or peripartum or peri-partum or parturition or puerperal or maternity or midwives or midwifery or birthing or childbearing or caesarean or cesarian or caesarean or caesarian or c-section* or episiotom* or endometritis) OR ((vagina* or placenta* or obstetric* or operative or procedur*2 or breech or pre-term or preterm or term or instrumental) and deliver*) OR ((delivery or labor or labour) and (pregnan* or neonatal or neo-natal or postnatal or post-natal or postpartum or post-partum or antenatal or ante-natal or antepartum or ante-partum or newborn or matern* or obstetric* or induction or inductive)))) AND (tw:((antibiotic* or anti-biotic* or antibacterial* or anti-bacterial* or amikacin* or Aminoglycoside* or aminopenicillin* or amoxicillin* or Amphenicol* or ampicillin* or ampictam* or augmentin* or azithromycin* or aztreonam* or benzathine* or Benzylpenicillin* or beta-lactam* or carbapenem* or carbenicillin* or cefaclor* or cefadroxil* or cefamandole* or cefazolin* or cefcapene* or cefclidine* or cefdaloxime* or cefditoren* or Cefepime* or cefetamet* or cefixime* or cefluprenam* or cefmenoxime* or cefodizime* or cefonicid* or cefoperazone* or ceforanide* or Cefotaxime* or cefotetan* or cefotiam* or Cefotoxime* or Cefoxitin* or cefozopran* or cefpimizole* or cefpirome* or cefpodoxime* or cefprozil* or cefquinome* or ceftazidime* or ceftibuten* or ceftizoxime* or Ceftolozane* or ceftriaxone* or cefuroxime* or cephalexin* or cephalosporin* or Cephalothin* or cephamycin* or cephapirin* or cephradine* or Chloramphenicol* or Ciprofloxacin* or clarithromycin* or clavamox* or clavulanate* or clavulanic acid* or clinafloxacin* or clindamicin* or clindamycin* or cloxacillin* or co-amoxyclav* or dicloxacillin* or doxycycline* or ertapenem* or Erythromycin* or Fluoroquinolone* or gatifloxacin* or gentamicin* or imipenem* or kanamycin* or levofloxacin* or Lincomycin* or Lincosamide* or lomefloxacin* or Macrolide* or maxifloxacin* or meropenem* or methicillin* or Metronidazole* or mezlocillin* or minocycline* or nafcillin* or Nitroimidazole* or norfloxacin* or ofloxacin* or oxacillin* or penicillin* or piperacillin* or procaine* or sparfloxacin* or Streptomycin* or sulbactam* or Tetracycline* or ticarcillin* or timentin* or tinidazol* or trovafloxacin* or tyclav* or unasyn*) OR (antiseptic* or anti-septic* or vaginal cleans* or skin prep* or saline wash or benzalkonium chloride* or carbolic acid spray* or cetrimide* or chlorhexidine* or gluconate* or hexachlorophene* or iodine* or iodophor* or isopropyl* or mercuric compound* or parachlorometaxylenol* or povidone-iodine* or triclosan* or vaginal metronidazole*))) AND (tw:(((sepsis or infection*) and (prophylaxis or prophylac* or prevent* or chemoprophylaxis)))) |
| --- |

## Maternity & Infant Care database search

Database(s): Maternity & Infant Care Database (MIDIRS) 1971 to 27 May 2022

| # | Searches |
| --- | --- |
| 1 | (obstetric* or childbirth* or birth*2 or intrapartum or intra-partum or antepartum or ante-partum or perinatal or peri-natal or peripartum or peri-partum or parturition or puerperal or maternity or midwives or midwifery or birthing or childbearing or cesar#an* or caesar#an* or c-section* or episiotom*).mp. |
| 2 | ((vagina* or placenta* or obstetric* or operative or procedur*2 or breech or pre-term or preterm or term or instrumental) adj3 deliver*).mp. |
| 3 | ((delivery or labor or labour) adj5 (pregnan* or neonatal or neo-natal or postnatal or post-natal or postpartum or post-partum or antenatal or ante-natal or antepartum or ante-partum or newborn or matern* or obstetric* or induction or inductive)).mp. |
| 4 | 1 or 2 or 3 |
| 5 | (antibiotic* or anti-biotic* or antibacterial* or anti-bacterial* or amikacin* or Aminoglycoside* or aminopenicillin* or amoxicillin* or Amphenicol* or ampicillin* or ampictam* or augmentin* or azithromycin* or aztreonam* or benzathine* or Benzylpenicillin* or beta-lactam* or carbapenem* or carbenicillin* or cefaclor* or cefadroxil* or cefamandole* or cefazolin* or cefcapene* or cefclidine* or cefdaloxime* or cefditoren* or Cefepime* or cefetamet* or cefixime* or cefluprenam* or cefmenoxime* or cefodizime* or cefonicid* or cefoperazone* or ceforanide* or Cefotaxime* or cefotetan* or cefotiam* or Cefotoxime* or Cefoxitin* or cefozopran* or cefpimizole* or cefpirome* or cefpodoxime* or cefprozil* or cefquinome* or ceftazidime* or ceftibuten* or ceftizoxime* or Ceftolozane* or ceftriaxone* or cefuroxime* or cephalexin* or cephalosporin* or Cephalothin* or cephamycin* or cephapirin* or cephradine* or Chloramphenicol* or Ciprofloxacin* or clarithromycin* or clavamox* or clavulanate* or clavulanic acid* or clinafloxacin* or clindamicin* or clindamycin* or cloxacillin* or co-amoxyclav* or dicloxacillin* or doxycycline* or ertapenem* or Erythromycin* or Fluoroquinolone* or gatifloxacin* or gentamicin* or imipenem* or kanamycin* or levofloxacin* or Lincomycin* or Lincosamide* or lomefloxacin* or Macrolide* or maxifloxacin* or meropenem* or methicillin* or Metronidazole* or mezlocillin* or minocycline* or nafcillin* or Nitroimidazole* or norfloxacin* or ofloxacin* or oxacillin* or penicillin* or piperacillin* or procaine* or sparfloxacin* or Streptomycin* or sulbactam* or Tetracycline* or ticarcillin* or timentin* or tinidazol* or trovafloxacin* or tyclav* or unasyn*).mp. |
| 6 | (antiseptic* or anti-septic* or vaginal cleans* or skin prep* or saline wash or benzalkonium chloride* or carbolic acid spray* or cetrimide* or chlorhexidine* or gluconate* or hexachlorophene* or iodine* or iodophor* or isopropyl* or mercuric compound* or parachlorometaxylenol* or povidone-iodine* or triclosan* or vaginal metronidazole*).mp. |
| 7 | 5 or 6 |
| 8 | 4 and 7 |
| 9 | ((sepsis or infection*) adj6 (prophylaxis or prophylac* or prevent*)).mp. |
| 10 | 8 and 9 |
| 11 | Puerperal infection.de. |
| 12 | 9 and 11 |
| 13 | 7 and 11 |
| 14 | (Antibiotic prophylaxis or "Antibiotics - therapeutic use").de. or Antibiotic Prophylaxis.mp. |
| 15 | infection control.mp. or Infection control.de. |
| 16 | antisepsis.mp. or Antisepsis.de. |
| 17 | asepsis.mp. |
| 18 | (infect* adj3 prevent*).mp. |
| 19 | 14 or 15 or 16 or 17 or 18 |
| 20 | 4 and 19 |
| 21 | 10 or 12 or 13 or 20 |
| 22 | limit 21 to yr="1990 -Current" |
| 23 | limit 22 to (animal study or briefing paper or case report or circular or circular letter or commentary or correspondence or editorial or fact sheet or historical article or interview or lecture or manual or news or news item or news release or newspaper clipping or product news) |
| 24 | 22 not 23 |

## CINAHL database search

| S1 | ( (MH “Delivery, Obstetric+/ae/pf/ct”) OR (MH “Surgery, obstetrical+”) OR (MH “cesarean section+”) OR (MH “obstetric care/pf/ae/ct”) OR (MH “perinatal care/ae/pf/ct”) OR (MH “labor+”) OR (MH “childbirth+”) OR (MH “management of labor”) OR (MH “labour complications”) OR (MH “midwifery+/pf”) OR (MH “Maternal-Child Nursing+/pf”) OR (MH “perinatology/pf”) OR (MH “obstetrics/pf”) OR (MH "Episiotomy") OR (MH "Vacuum Extraction, Obstetrical") OR (MH "Vaginal Birth+") ) AND ( (MH “Sepsis+/pc”) OR (MH “wound infection/pc”) OR MH “infection/pc”) OR (MH “Endometrial Diseases/pc”) OR (MH “Surgical Wound Infection+/pc/dt/pf”) OR (MH “perioperative care+”) ) AND ( (MH “Antiinfective agents”) OR (MH “antibiotics+”) OR (MH “Antiinfective Agents, Local+”) OR (MH “Antiinfective Agents, Quinolone+”) ) |
| --- | --- |
| S2 | ( ( (MH “puerperal infection/pc”) OR (MH “Pregnancy Complications, Infectious/pc”) ) ) AND ( ( (MH “Antiinfective agents”) OR (MH “antibiotics+”) OR (MH “Antiinfective Agents, Local+”) OR (MH “Antiinfective Agents, Quinolone+”) ) OR ( (MH “antibiotic prophylaxis+”) ) ) |
| S3 | ( ( (MH “puerperal infection”) OR (MH “Pregnancy Complications, Infectious”) ) ) AND ( ( (MH “Sepsis+/pc”) OR (MH “wound infection/pc”) OR MH “infection/pc”) OR (MH “Endometrial Diseases/pc”) OR (MH “Surgical Wound Infection+/pc/dt/pf”) OR (MH “perioperative care+”) ) OR ( (MH “Antiinfective agents”) OR (MH “antibiotics+”) OR (MH “Antiinfective Agents, Local+”) OR (MH “Antiinfective Agents, Quinolone+”) ) OR ( (MH “antibiotic prophylaxis+”) ) ) |
| S4 | ( (MH “antibiotic prophylaxis+”) ) AND ( ( (MH “Delivery, Obstetric+/ae/pf/ct”) OR (MH “Surgery, obstetrical+”) OR (MH “cesarean section+”) OR (MH “obstetric care/pf/ae/ct”) OR (MH “perinatal care/ae/pf/ct”) OR (MH “labor+”) OR (MH “childbirth+”) OR (MH “management of labor”) OR (MH “labour complications”) OR (MH “midwifery+/pf”) OR (MH “Maternal-Child Nursing+/pf”) OR (MH “perinatology/pf”) OR (MH “obstetrics/pf”) OR (MH "Episiotomy") OR (MH "Vacuum Extraction, Obstetrical") OR (MH "Vaginal Birth+") ) ) |
| S5 | S1 or S2 or S3 or S4 |
| S6 | ( (obstetric* or childbirth* or birth*2 or intrapartum or intra-partum or antepartum or ante-partum or perinatal or peri-natal or peripartum or peri-partum or parturition or puerperal or maternity or midwives or midwifery or birthing or childbearing or cesar#an* or caesar#an* or c-section* or episiotom*) ) OR ( (vagina* or placenta* or obstetric* or operative or procedur*2 or breech or pre-term or preterm or term or instrumental) n2 (deliver*) ) OR ( ( (delivery or labor or labour) n4 (pregnan* or neonatal or neo-natal or postnatal or post-natal or postpartum or post-partum or antenatal or ante-natal or antepartum or ante-partum or newborn or matern* or obstetric* or induction or inductive) ) ) |
| S7 | ( (antibiotic* or anti-biotic* or antibacterial* or anti-bacterial* or amikacin* or Aminoglycoside* or aminopenicillin* or amoxicillin* or Amphenicol* or ampicillin* or ampictam* or augmentin* or azithromycin* or aztreonam* or benzathine* or Benzylpenicillin* or beta-lactam* or carbapenem* or carbenicillin* or cefaclor* or cefadroxil* or cefamandole* or cefazolin* or cefcapene* or cefclidine* or cefdaloxime* or cefditoren* or Cefepime* or cefetamet* or cefixime* or cefluprenam* or cefmenoxime* or cefodizime* or cefonicid* or cefoperazone* or ceforanide* or Cefotaxime* or cefotetan* or cefotiam* or Cefotoxime* or Cefoxitin* or cefozopran* or cefpimizole* or cefpirome* or cefpodoxime* or cefprozil* or cefquinome* or ceftazidime* or ceftibuten* or ceftizoxime* or Ceftolozane* or ceftriaxone* or cefuroxime* or cephalexin* or cephalosporin* or Cephalothin* or cephamycin* or cephapirin* or cephradine* or Chloramphenicol* or Ciprofloxacin* or clarithromycin* or clavamox* or clavulanate* or clavulanic acid* or clinafloxacin* or clindamicin* or clindamycin* or cloxacillin* or co-amoxyclav* or dicloxacillin* or doxycycline* or ertapenem* or Erythromycin* or Fluoroquinolone* or gatifloxacin* or gentamicin* or imipenem* or kanamycin* or levofloxacin* or Lincomycin* or Lincosamide* or lomefloxacin* or Macrolide* or maxifloxacin* or meropenem* or methicillin* or Metronidazole* or mezlocillin* or minocycline* or nafcillin* or Nitroimidazole* or norfloxacin* or ofloxacin* or oxacillin* or penicillin* or piperacillin* or procaine* or sparfloxacin* or Streptomycin* or sulbactam* or Tetracycline* or ticarcillin* or timentin* or tinidazol* or trovafloxacin* or tyclav* or unasyn*) ) OR ( (antiseptic* or anti-septic* or vaginal cleans* or skin prep* or saline wash or benzalkonium chloride* or carbolic acid spray* or cetrimide* or chlorhexidine* or gluconate* or hexachlorophene* or iodine* or iodophor* or isopropyl* or mercuric compound* or parachlorometaxylenol* or povidone-iodine* or triclosan* or vaginal metronidazole*) ) |
| S8 | ( (sepsis or infection*) n5 (prophylaxis or prophylac* or prevent*) ) |
| S9 | S6 and S7 and S8 |
| S10 | S5 or S9 |
| - | *Manually add limiter for date: 1995-2022* |
