# Supplementary material for: Factors affecting the use of antibiotics and antiseptics to prevent maternal infection at birth: A global mixed-methods systematic review
Source: PLoS One. 2022 Sep 1;17(9):e0272982. doi: 10.1371/journal.pone.0272982 (PMC9436089; doi:10.1371/journal.pone.0272982)
Supplement: S1 Table — (DOCX) [file pone.0272982.s003.docx]

**S1 Table. CASP assessments of qualitative and mixed methods studies**

| **Lead author and year** | **Statement of the aims of the research** | **Was a qualitative methodology appropriate? ^a^** | **Appropriate research design ^a^** | **Appropriate recruitment strategy ^a^** | **Adequate consideration of reflexivity** | **Have ethical issues been taken into consideration?** | **Sufficiently rigorous data analysis** | **Were the findings supported by the evidence?** | **How valuable is the research?** | **Overall concerns about methodological limitations** |
| --- | --- | --- | --- | --- | --- | --- | --- | --- | --- | --- |
| **Berrow 1997** | Yes (p.182) | Yes | Yes | Unclear - Not specified | No - No reflexive statement | Unclear – No mention of ethics approval or informed consent | Partial - Appears to be thematic analysis; minimal detail on methods | Unclear - Minimal discussion of relevant examples | Somewhat valuable | Serious concerns |
| **Everitt 1990** | Yes (p.579) | Yes | Unclear - Insufficient information given | Unclear - Not specified | No - No reflexive statement | Unclear – No mention of ethics approval or informed consent | Unclear - Not described | Unclear - Evidence presented is minimal | A little valuable | Serious concerns |
| **Høgh-Poulsen 2021** | Yes (p.65 | Yes | Yes | Yes | Partial – researchers background was mentioned yet not tied to reflexive process | Unclear – consent retrieved yet no ethics approval mentioned | Yes | Yes | Valuable | Minor concerns |
| **Kolkman 2017** | Yes (p.3) | Yes | Yes | Partial - Some information about recruitment but unclear how OBS and microbiologists recruited or selected | No – No reflexive statement | Yes | Yes | Yes | Somewhat valuable | Minor concerns |
| **Liabsuetrakul 2002^41^ & 2003*^b^*** | Yes (p.263; p.1667) | Yes | Yes | Yes | Yes | Yes | Partial - Minimal description in 2002 paper | Yes | Valuable | No or very minor concerns |
| **Sumankuuro 2018** | Yes (p.3) | Yes | Yes | Yes | Yes | Yes | Yes | Yes | Somewhat valuable | No or very minor concerns |
| **Weckesser 2019** | Yes (p.2) | Yes | Yes | Yes | Yes | Yes | Yes | Yes | Valuable | No or very minor concerns |

*^a^ appropriate given the stated aims of the research; ^b^ two papers for one study*
