# Supplementary material for: Factors affecting the use of antibiotics and antiseptics to prevent maternal infection at birth: A global mixed-methods systematic review
Source: PLoS One. 2022 Sep 1;17(9):e0272982. doi: 10.1371/journal.pone.0272982 (PMC9436089; doi:10.1371/journal.pone.0272982)
Supplement: S2 Table — (DOCX) [file pone.0272982.s004.docx]

## S2 Table: Newcastle-Ottawa Scale assessments of studies with quantitative methods

| **Lead author and year** | **Selection (Max. 5)** | **Comparability  (Max. 2)** | **Outcomes Measures & Analysis  (Max. 3)** | **Total score (Max. 10)** | **Classification** |
| --- | --- | --- | --- | --- | --- |
| **Brisibe 2014** | 3 | 2 | 2 | 7 | Good |
| **Edwards 2015** | 1 | 2 | 2 | 5 | Satisfactory |
| **Everitt 1990** | 4 | 0 | 2 | 6 | Satisfactory |
| **Goldstick 2005** | 3 | 2 | 2 | 7 | Good |
| **Jakes 2020** | 2 | 0 | 2 | 4 | Unsatisfactory |
| **Konrad 2007** | 4 | 0 | 2 | 6 | Satisfactory |
| **Liabsuetrakul 2002** | 4 | 2 | 2 | 8 | Good |
| **Liabsuetrakul 2003** | 5 | 2 | 2 | 9 | Good |
| **Muthukumarappan 2000** | 4 | 0 | 2 | 6 | Satisfactory |
| **Price 2018** | 1 | 2 | 2 | 5 | Satisfactory |
| **Raghunathan 2013** | 4 | 2 | 2 | 8 | Good |
| **Rambourdin 2013** | 3 | 0 | 1 | 4 | Unsatisfactory |
| **Tully 2002** | 3 | 2 | 1 | 6 | Satisfactory |
| **Watson 2019** | 3 | 1 | 2 | 6 | Satisfactory |
| **Watt 2001** | 3 | 2 | 2 | 7 | Good |
