# Supplementary material for: Factors affecting the use of antibiotics and antiseptics to prevent maternal infection at birth: A global mixed-methods systematic review
Source: PLoS One. 2022 Sep 1;17(9):e0272982. doi: 10.1371/journal.pone.0272982 (PMC9436089; doi:10.1371/journal.pone.0272982)
Supplement: S3 Table — (DOCX) [file pone.0272982.s005.docx]

**S3 Table. GRADE-CERQual evidence profile**

| **Themes and summary of review finding** | **Contributing studies** | **Methodological limitations assessment** | **Coherence assessment** | **Relevance assessment** | **Adequacy assessment** | **Overall GRADE-CERQual assessment and explanation** |
| --- | --- | --- | --- | --- | --- | --- |
| Providers have mixed views on whether prophylactic antibiotics are effective and beneficial for preventing infection. | 17,34,41 | **Minor concerns:** 1 study with no or minor concerns  1 study with serious concerns (recruitment, reflexivity, ethics, data analysis and support for findings) | **No or very minor concerns** | **Serious concerns:**  Two countries (England, Thailand). Two income level settings (Upper middle and high). One study with indirectly relevant aims. | **Serious concerns:** Two studies, one moderately thick and one thin data. | **Low confidence:**  Minor concerns about methodological limitations. Serious concerns about relevance (upper-middle to high income countries from two regions) and adequacy (two studies). |
| Some physicians are more likely to use antibiotics for high-risk women undergoing caesarean section or following complications during the procedure, and less likely to prescribe for women undergoing elective caesarean section. Others use antibiotics routinely for all women undergoing caesarean section. | 17,41 | **No or very minor concerns** | **No or very minor concerns** | **Serious concerns:**  One study from Thailand. Upper middle income country setting. | **Serious concerns:** 1 study with moderately thick data | **Very low confidence:**  Serious concerns about relevance (upper-middle income country in one region) and adequacy (one study). |
| Some providers are concerned about unnecessary antibiotic use due to potential for unwanted side effects, overtreatment and medicalisation of birth, while others consider adverse reactions are low and outweighed by harm from infection. | 17,39,41 | **Minor concerns: 1 study with no or very minor concerns**  **1 study with minor concerns (recruitment, reflexivity)** | **No or very minor concerns** | **Serious concerns:**  Two countries (Netherlands, Thailand). Two income level settings (Upper middle and high income). One study with indirectly relevant aims. | **Serious concerns:** Two studies with thin data | **Low confidence:**  Minor concerns regarding methodological limitations. Serious concerns about relevance (upper-middle to high income countries from two regions) and adequacy (two studies). |
| Providers have varying levels of concern about antimicrobial resistance - some prescribe less antibiotics for this reason, while others consider it is not a threat and have not changed their antibiotic prescription practice. | 17,34,39 | **Moderate concerns:** 1 study with no or very minor concerns  1 study with minor concerns (recruitment, reflexivity)  1 study with serious concerns (recruitment, reflexivity, ethics, data analysis, support for findings) | **No or very minor concerns** | **Serious concerns:**  Three countries (Netherlands, England, Thailand). Two income level settings (Upper middle and high income). Two studies with indirectly relevant aims. | **Serious concerns:** Two studies with moderately thick and one with thin data. | **Low confidence:** Moderate concerns about methodological limitations. Serious concerns about relevance (upper-middle income countries in two regions) and adequacy (three studies). |
| Some physicians are motivated by a fear of post-operative infection, and the risk of resulting blame and damage to their professional reputation. This can lead to a belief that erring on the side of overtreatment is preferable to undertreatment. | 17,41 | **No or very minor concerns:** 1 study with no or very minor concerns | **No or very minor concerns** | **Serious concerns:** One study from Thailand. Upper middle income country setting. | **Serious concerns:** 1 study with moderately thick data | **Very low confidence:**  Serious concerns about relevance (upper-middle income country in one region) and adequacy (one study). |
| The risk of infection, and therefore the need for antibiotics, is considered by some providers to vary depending on local environmental factors. | 34,41 | **Minor concerns:** 1 study with no or minor concerns  1 study with serious concerns (recruitment, reflexivity, ethics, data analysis and support for findings) | **No or very minor concerns** | **Serious concerns:**  Two countries (England, Thailand). Two income level settings (Upper middle and high). One study with indirectly relevant aims. | **Serious concerns:** Two studies, one moderately thick and one thin data. | **Low confidence:**  Moderate concerns about methodological limitations. Serious concerns about relevance (upper-middle to high income countries in two regions) and adequacy (two studies). |
| Providers' choice of a particular antibiotic agent is informed by whether it is recommended or common practice and perceptions of its effectiveness relative to other options. | 37,41 | **Moderate concerns:** 1 study with no or very minor concerns  1 study with serious concerns (research design, recruitment, reflexivity, ethics, data analysis, support for findings) | **No or very minor concerns** | **Serious concerns:** Two countries (Thailand, USA). Two income levels (upper middle and high income). One study with indirectly relevant aims. | **Serious concerns:** One study with thin data, one with moderately thick data. | **Low confidence:**  Moderate concerns about methodological limitations. Serious concerns about relevance (upper-middle to high income countries frm two settings) and adequacy (two studies). |
| Providers are influenced by locally recommended practices and personal experience in deciding how many doses to prescribe, with some believing multiple dose regimens are more effective. | 17,41 | **No or very minor concerns** | **No or very minor concerns** | **Serious concerns:**  One study from Thailand. Upper middle income country setting. | **Serious concerns:** 1 study with moderately thick data | **Very low confidence:**  Serious concerns about relevance (upper-middle income country in one region) and adequacy (one study). |
| Providers generally commence antibiotic administration after clamping the umbilical cord, with reasons including avoiding passing antimicrobial agents to the baby or in response to complications or potential contamination during surgery. | 37,41 | **Moderate concerns:** 1 study with no or very minor concerns. 1 study with serious concerns (research design, recruitment, reflexivity, ethics, data analysis, support for findings) | **No or very minor concerns** | **Serious concerns:** Two countries (Thailand, USA). Two income levels (upper middle and high income). One study with indirectly relevant aims. | **Serious concerns:** Two studies with thin data. | **Low confidence:**  Moderate concerns about methodological limitations.  Serious concerns about relevance (upper-middle income country in two regions) and adequacy (two studies). |
| Providers may have regard to the cost-effectiveness and affordability of antibiotics when deciding whether to prescribe and in choosing a particular antibiotic agent. | 17,41,46 | **No or very minor concerns** | **No or very minor concerns** | **Serious concerns:** Two countries (Thailand, Ghana). Two income level settings (lower middle and upper middle). One study with indirectly relevant aims. | **Serious concerns:** One study with moderately thick data and one with thin data. | **Low confidence:** Serious concerns about relevance (middle income countries in two regions) and adequacy (two studies). |
| Some consider that the evidence regarding prophylactic antibiotics is not applicable to their local setting. They express a preference for evidence from local trials. | 17,34 | **Minor concerns:** 1 study with no or minor concerns  1 study with serious concerns (recruitment, reflexivity, ethics, data analysis and support for findings) | **No or very minor concerns** | **Serious concerns:**  Two countries (England, Thailand). Two income level settings (Upper middle and high). One study with indirectly relevant aims. | **Serious concerns:** Two studies, one moderately thick and one thin data. | **Low confidence:** Minor concerns about methodological limitations. Serious concerns about relevance (upper-middle income countries in two regions) and adequacy (two studies). |
| Providers obtain knowledge regarding appropriate antibiotic prescribing practices from varying sources. There are mixed views on the usefulness and uptake of guidelines. Some providers express preference for textbooks over journals. | 17,41,46 | **No or very minor concerns** | **No or very minor concerns** | **Serious concerns:** Two countries (Thailand, Ghana). Two income level settings (lower middle and upper middle). One study with indirectly relevant aims. | **Serious concerns:** One study with moderately thick data and one with thin data. | **Low confidence:**  Serious concerns about relevance (middle income countries in two regions) and adequacy (two studies). |
| Some providers antibiotic prescribing practices were highly influenced by professional norms and expectations, including pressure from colleagues and the observed practice of supervisors. | 17,41 | **No or very minor concerns** | **No or very minor concerns** | **Serious concerns:**  One study from Thailand. Upper middle income country setting. | **Serious concerns:** 1 study with moderately thick data | **Very low confidence:**  Serious concerns about relevance (upper-middle income country in one region) and adequacy (one study). |
